# Vitamin C in the Management of Thyroid Cancer: A Highway to New Treatment?

**DOI:** 10.3390/antiox13101242

**Published:** 2024-10-15

**Authors:** Francesca Gorini, Alessandro Tonacci

**Affiliations:** Institute of Clinical Physiology, National Research Council, 56124 Pisa, Italy; alessandro.tonacci@cnr.it

**Keywords:** thyroid cancer, vitamin C, ascorbate, antioxidants, oxidative stress, reactive oxygen species, cancer management, sensory analysis

## Abstract

Thyroid cancer (TC) is the most common endocrine malignancy, with an increased global incidence in recent decades, despite a substantially unchanged survival. While TC has an excellent overall prognosis, some types of TC are associated with worse patient outcomes, depending on the genetic setting. Furthermore, oxidative stress is related to more aggressive features of TC. Vitamin C, an essential nutrient provided with food or as a dietary supplement, is a well-known antioxidant and a scavenger of reactive oxygen species; however, at high doses, it can induce pro-oxidant effects, acting through multiple biological mechanisms that play a crucial role in killing cancer cells. Although experimental data and, less consistently, clinical studies, suggest the possibility of antineoplastic effects of vitamin C at pharmacological doses, the antitumor efficacy of this nutrient in TC remains at least partly unexplored. Therefore, this review discusses the current state of knowledge on the role of vitamin C, alone or in combination with other conventional therapies, in the management of TC, the mechanisms underlying this association, and the perspectives that may emerge in TC treatment strategies, and, also, in light of the development of novel functional foods useful to this extent, by implementing novel sensory analysis strategies.

## 1. Introduction

Thyroid cancer (TC), the most common endocrine neoplasm, represents around 1% of malignant cancers [1]. In the last decades, global TC incidences have continuously increased and in 2020, approximately 586,000 cases of TC were reported worldwide, making TC among the 10 most frequently diagnosed cancers and with a female-to-male incidence ratio of 3:1 (10.1 per 100,000 women and 3.1 per 100,000 men) [2,3,4]. Of note, the incidence of TC can vary widely by geographic location, with the highest incidence detected in both high- and middle-income countries (e.g., Italy, France, Austria, United States—US, Canada, Republic of Korea, Brazil, China, Costa Rica) probably due to a greater possibility of access to diagnostics, which also suggests a non-negligible effect of overdiagnosis on TC epidemiology in many world areas [3,5]. In contrast, mortality rates appear relatively similar across settings although they differ by sex, with deaths recorded among women almost double those among men in 2020 (27,700 vs. 15,900) [2,3]. The prognosis of TC is generally excellent, and mortality is very low compared with the incidence (between 0.3 and 0.5 deaths per 100,000 per year) [3,5]. In particular, tumors originally known as differentiated thyroid cancers (DTC), which account for more than 95% of cases, have a 10-year survival rate of over 90% [6,7]. According to the fifth edition of the Classification of Endocrine and Neuroendocrine Tumors released by the World Health Organization (WHO) in 2022, thyroid neoplasms arising from follicular cells are divided into three categories—benign, low-risk, and malignant neoplasms—which better reflect molecular and histopathological characteristics, biological behavior, and prognostic risk [8,9]. In the new WHO classification, greater attention has been paid to the histological subtyping of papillary thyroid carcinoma (PTC), the most frequent thyroid malignancy, whose typical hallmarks are point mutations or gene rearrangements involving the mitogen-activated protein kinase/extracellular signal-regulated kinase (MAPK/ERK) pathway, which regulates cell proliferation, differentiation, migration, senescence, and apoptosis [8,9,10]. Although PTCs rarely behave as aggressive tumors, a point mutation in v-raf murine sarcoma viral oncogene homolog B1 (*BRAF*) (encoding a serine-threonine kinase that is a constituent of the MAPK pathway), namely, *BRAF V600E*, which accounts for 30–70% of mutations in all PTCs, has been linked to extra-thyroidal extension and lymph node metastasis [8,11,12,13]. *BRAF V600E* mutation is also frequently found in patients with anaplastic thyroid cancer (ATC), the fastest-growing and most aggressive type of undifferentiated TC, and has been associated with a poor prognosis [14].

While thyroid hormones (TH) physiologically have a substantial impact on cellular redox balance due to their role in cellular metabolism and oxygen consumption, excessive production of reactive oxygen species (ROS) can lead to DNA damage and initiate the process of carcinogenesis and maintain genomic instability during the later stages of TC, with a relationship between ROS levels and tumor aggressiveness [15,16,17]. Furthermore, oxidative stress can be partly responsible for *BRAF* mutations and, on the other hand, *BRAF V600E* can upregulate the nicotinamide adenine dinucleotide phosphate (NADPH) oxidase 4 (NOX4), which generates ROS in a variety of tissues and is highly expressed in numerous tumors including PTC [16,18,19].

Vitamin C, existing in the two main forms of ascorbic and dehydroascorbic acid, is an essential nutrient for humans, acting as a cofactor for several enzymes and antioxidants and, as such, is involved in the primary prevention of complex conditions such as coronary heart disease, stroke, and cancer [20,21,22]. If, at physiological micromolar concentrations, ascorbate acts as an antioxidant, reducing ROS levels, pharmacological doses of vitamin C (reaching millimolar plasma concentrations) can generate pro-oxidant effects, mediated by the accumulation of hydrogen peroxide (H_2_O_2_), thereby killing cancer cells in vitro and slowing tumor growth in vivo [21,23]. While certain data from clinical studies support the role of vitamin C as a potent antitumor agent when administered intravenously and at high doses in various cancer types and some hypotheses on the anticancer mechanisms have been generated [23,24], whether vitamin C is also able to impact TC remains to be fully clarified [25]. In this review, we have summarized the current knowledge on the potential role of vitamin C in the treatment of TC, discussing the possible mechanisms underlying the antitumor activities of ascorbate and the potential perspectives arising from the use of vitamin C alone or in combination with standard therapies in the management of this endocrine neoplasm, also focusing on the enhancement of the acceptability of vitamin C intake through food by the end users.

## 2. Thyroid Cancer: Epidemiology and Classification

Most of the global increase in incidence of TC is attributable to PTC, which represents the most common malignancy deriving from follicular cells in both adults and children [9,26]. As highlighted in several reports, the more widespread use of medical imaging techniques such as thyroid ultrasonography has allowed a timely detection of nodules as small as 2 mm, while needle biopsy procedures have improved the identification of both malignant cancers and indolent tumors that are irrelevant to patient’s health, overall leading to the so-called “epidemic of overdiagnosis” in nearly every region of the world [5,26,27]. On the other hand, an increase in the number of all sizes of PTC, including those exceeding 1 cm, was also documented [26,27]. A recent analysis reported that in the US, the overall PTC incidence rate increased from 9.9 to 16.1 per 100,000 between 2003 and 2017, but after reaching a maximum in 2015, it began to follow a downward trend [28]. These data were confirmed in a subsequent trend analysis that observed a decreasing trend in the incidence of TC from 2014 to 2019 in the US, with the largest reduction in annual percentage changes in individuals with all TC and PTC in 55–69 age groups, women, Black and non-Hispanic ethnicities, the highest socioeconomic status groups, and urban regions [29]. The decreasing trend detected in recent years in the US is attributed to the promotion of healthy lifestyles (decrease in obesity, overweight, smoking, alcohol, and iodine intake) and the reduction of exposure to environmental risk factors (ionizing radiation and endocrine disrupting chemicals) but above all to changes in clinical practice guidelines for the management of TC over the last 15 years [5,29]. Indeed, the latest guidelines of the American Thyroid Association no longer recommend fine-needle biopsy for micropapillary TCs in the absence of evidence of extrathyroidal extension, metastatic cervical lymph nodes, or distant metastases [30]. In addition, the reclassification of the noninvasive encapsulated follicular variant PTC (EFV-PTC) with a low risk of adverse outcomes to “noninvasive follicular thyroid neoplasm with papillary-like nuclear features” (NIFTP), an encapsulated and noninvasive tumor with an excellent prognosis, has further contributed to the recent decline in total PTC incidence [5,31].

PTCs, which are divided into eight subtypes based on the histological definition and molecular profiles, encompass the infiltrative growth pattern, while the invasive encapsulated follicular variant of PTC (IEFV-PTC) is no longer included in the PTC category and is considered a separate group within well-differentiated TC, with generally a better prognosis than infiltrative PTCs [8,9,32]. Furthermore, the latest WHO guidelines recommend that PTCs measuring ≤1 cm (called papillary microcarcinomas), which generally show an indolent behavior and rarely progress, should be classified based on histomorphology features and not as a distinct subtype [9,32]. *BRAF* mutations are associated with worse outcomes in PTC subtypes, such as tall cell, columnar cell, and hobnail subtypes, which present with aggressive clinicopathological features (discussed in the next section) [8,9]. Follicular thyroid carcinoma (FTC), mostly characterized by alterations in the rat sarcoma gene (*RAS*, encoding another component of the MAPK cascade) and histologically stratified into three different subtypes reflecting prognosis, is also included within the category of well-differentiated TCs [8]. IEFV-PTC presents certain nuclear characteristics of PTC in the infiltrative subtype; however, in the incapsulated form, it is divided into three subtypes that correlate the state of invasion to the prognosis, similar to FTC [8]. The invasive neoplasms known as “oncocytic thyroid carcinomas”, divided into three subtypes and composed of at least 75% oncocytic cells, do not show dedifferentiation like high-grade TC but are characterized by an increased mitotic activity (≥5 mitoses per 2 mm^2^) and tumor necrosis [8,32]. The 2022 WHO classification also introduced new categories of high-grade non-anaplastic malignant tumors with intermediate prognosis, including poorly differentiated thyroid carcinoma, presenting >3 mitoses per 2 mm^2^ and/or tumor necrosis and aberrant *RAS* signaling related to FTC; differentiated high-grade carcinoma, which has tumor necrosis and/or ≥5 mitoses per 2 mm^2^ as specific features, and, in the majority of cases, *BRAF V600E*-driven as it displays the cytoarchitectural properties of PTC [8,9,32]. Mutation in the promoter of telomerase reverse transcriptase (*TERT*), which is associated with telomere length and risk of several cancers [33] and in the p53 tumor suppressor (*TP53*), a gene that has a crucial role in maintaining genetic stability and preventing cancer development [34], have been detected in the dedifferentiation process and, therefore, are generally linked to aggressive behaviors and poor prognosis [8,9]. ATC, the most aggressive thyroid malignancy characterized by a predominant loss of differentiation, has as its hallmark the expression of *BRAF V600E* in the almost totality of cases, while three-quarters of cases harbor a differentiated TC (typically high-grade PTC) from which it probably originates [9] (Table 1).

### 2.1. Genetic Setting in Thyroid Cancer

Genetic predisposition is one of the major risk factors for the development of TC [35]. Thanks to the remarkable progress in gene sequencing techniques achieved in the last three decades, TC-associated genetic abnormalities (detected in more than 90% of patients) have been identified [36,37].

It is well established that TC generally involves genetic changes that occur in genes encoding proteins of the MAPK/ERK and phosphatidylinositol-3 kinase/protein kinase B (PI3K/AKT) signaling pathways [35,36]. While MAPK/ERK cascade regulates processes such as cell proliferation, differentiation, apoptosis, and stress responses and plays a key role in PTC initiation through point mutations of *BRAF* and *RAS* and *RET/PTC* and *TRK* rearrangements [36,37,38], PI3K/AKT is an oncogenic pathway controlling multiple aspects of cancer onset and progression, including cell survival, glucose metabolism, metastasis, and angiogenesis and is possibly implicated in FTC development by activating mutations in *RAS*, *PI3KCA*, and *AKT1* and inactivating *PTEN* [36,37,39]. Most of the genetic alterations in TC—point mutations and chromosomal rearrangements—are non-inherited and originate directly in the thyroid tissue [35,37]. Although these genetic aberrations are generally mutually exclusive with occurrence varying in the different TCs, concomitant mutations of *RET/PTC*, *RAS*, or *BRAF* have been reported in PTC in association with advanced stage of disease and poor prognosis [40,41,42]. Inherited mutations in the *RET* gene are instead associated exclusively with familial forms of medullary thyroid carcinoma (MTC), which, unlike other malignant thyroid neoplasms, originates from the parafollicular cells of the thyroid gland and with multiple endocrine neoplasia syndrome (MEN2A and MEN2B)-inherited diseases that lead to further abnormal activation of one or more endocrine glands [43,44,45]. The following subsections provide brief descriptions of the main features of the most common TC-related gene alterations.

#### 2.1.1. *RAS*

*RAS*, the most frequently varied gene in human cancer and the second in thyroid nodules [46], encodes guanosine triphosphate (GTP)-binding proteins, acts upstream BRAF and transmits a mitogen signal from the transmembrane tyrosine kinase membrane receptor to the nucleus via effectors in the MAPK and PI3K-AKT signaling pathways [35,37]. Point mutations of *RAS* variants, i.e., *HRAS*, *KRAS*, and *NRAS*, have been detected at various steps of tumorigenesis leading to TC, with a higher frequency in poorly differentiated and undifferentiated TC [36,47,48]. Indeed, RAS activation leads to DNA damage and dedifferentiation by inhibiting TTF-1 and PAX8, which have a central role in maintaining thyroid differentiation as they control the expression of genes for thyroglobulin (Tg), thyroperoxidase (TPO), and sodium/iodide symporter (NIS) involved in the function of thyroid follicular cells, with a correlation between the extent of *RAS* oncogene expression and the loss of thyroid differentiated phenotype [48,49]. Importantly, RAS-induced cell proliferation is positively associated with absent or low levels of thyroid stimulating hormone (TSH) [40]. Based on the latest published data, *RAS* mutations have been reported in up to 68% of cases with FTC, 20–40% with ATC, and 10–30% with PTC [35,37,48] (Table 2).

#### 2.1.2. *BRAF*

BRAF is a serine-threonine protein kinase belonging to the RAF family, which, once activated by RAS, induces MEK, resulting in the activation of downstream effectors of the MAPK pathway [36]. Pathogenic *BRAF* mutations, which lead to the constitutive activation of the MPAK signaling cascade, are present in approximately 4% of all cancers, with the substitution of valine for glutamate at codon 600—*BRAF V600E*—accounting for at least 56% of all *BRAF* mutations and 70% of all PTCs [35,50,51]. The incidence of *BRAF V600E* may vary depending on the PTC subtype (with a higher frequency in the aggressive tall cell subtype, up to 100% of cases, [52]) and different populations (ranging from approximately 20 to 75%, with *BRAF V600E* considered a major driver of PTC in Chinese PTC populations) [53]. In addition to PTC, *BRAF V600E* has been detected exclusively in ATC in proportions ranging from 20 to 45% [35,37]. Albeit with conflicting results, *BRAF V600E* mutations have been shown to be a sensitive marker for aggressive TCs [9,54]. A recent meta-analysis reported a significantly increased risk of multifocality, extrathyroidal invasion, local and distant lymph node metastases, recurrence, and decreased 10-year survival in papillary microcarcinomas carrying the *BRAF V600E* mutation [55] (Table 2).

#### 2.1.3. *RET/PTC*

Unlike germline and somatic point mutations in the rearranged during transfection (*RET*) proto-oncogene, encoding a transmembrane receptor-type tyrosine kinase that stimulates both MAPK/ERK and PIP3/AKT pathways and is responsible for the development of most cases of MEN2 and sporadic MTC, respectively, chromosomal rearrangements generated by the fusion of the C-terminal kinase region of *RET* (primarily expressed in thyroid parafollicular cells) with the N-terminal end of heterogeneous genes carrying a promoter for expression in thyroid follicular cells allow for the dimerization motif that determines the constitutive activation of RET kinase [36,40,56,57]. Among the different forms of *RET* rearrangement (around two dozen) identified as a PTC-specific genetic event, *RET/PTC1* and *RET/PTC3* are the most common, representing up to 90% of all *RET/PTC* rearrangements, *RET/PTC1* 2-fold being more frequent than *RET/PTC3* [58,59]. The mean prevalence of *RET/PTC* is around 20% although it can present great variability, up to 70–80% in atomic bomb survivors and in post-Chernobyl thyroid tumors [60,61]. In fact, *RET/PTC3* rearrangement is more prevalent in patients with a history of radiation exposure and in young adults (age < 18 years), while the female sex is associated with a higher prevalence of *RET/PTC1* in non-irradiated subjects [60,61] (Table 2).

#### 2.1.4. *PAX8/PPARγ*

The *PAX8/PPARγ* rearrangement, identified in 12–60% of FTC and up to 16% of PTC cases, consists of a stable translocation between the promoter and the majority of paired box gene 8 (*PAX8*) and the coding exons of peroxisome proliferator-activated receptor gamma (*PPARγ*), a member of the steroid/thyroid nuclear receptor family, which is normally expressed at very low levels in thyroid gland and probably exerts effects as tumor suppressor gene in thyroid and non-thyroidal cell lines [35,37,62,63,64]. The fusion protein, known as PPFP, may act as an oncoprotein, accelerating cell growth rates and DNA synthesis and reducing the rate of apoptosis [63]. Furthermore, PPFP appears to inhibit wild-type PPARγ functioning and stimulate or suppress PAX8-responsive genes [63]. Notably, most FTCs develop from two mutually exclusive pathways: one involving *RAS* mutation and the other manifesting *PAX8/PPARγ* rearrangement, and the latter is considered a distinct entity in TC as it is more prevalent in younger patients and associated with vascular invasion and capsular penetration [65,66] (Table 2).

#### 2.1.5. *PTEN*

Phosphatase and tensin homolog (*PTEN*) is the major negative regulator of the PI3K/AKT pathway, which, once activated by the binding of RAS or other growth factors to the catalytic subunit of PI3K, phosphorylates AKT, which, in turn, activates downstream effectors, including mammalian target of rapamycin (mTOR) [40]. Loss of heterozygosity of germline mutations of *PTEN*, which are inherited with an autosomal dominant pattern, leads to the development of various types of benign and malignant tumors, including TC, a condition known as hamartoma tumor syndrome (PHTS) [67]. The incidence of TC in patients with PHTS ranges from 4 to 33% in PDTC, 11 to 20% in ATC, and lower frequencies in advanced DTC (up to 14% in FTC) [68]. Conversely, although somatic *PTEN* mutations can be detected in thyroid nodules, often associated with follicular patterns, their characteristics and role as prognostic indicators still need to be elucidated [40,68] (Table 2).

#### 2.1.6. *TERT*

*TERT* encodes telomerase reverse transcriptase, the catalytic subunit of the enzyme telomerase, which plays a critical role in most human cancers by ensuring telomere length and subsequent chromosomal stability and preventing cellular senescence [38,69]. Among the point mutations in the *TERT* promoter, responsible for the increased gene transcription, cytosine to thymine transitions C228T and C250T are the most common, with the former more prevalent in TC [33,70]. *TERT* mutations can be detected in around 10–15% of PTCs and 17% of patients with FTC but are present with higher frequencies in aggressive or undifferentiated tumors [37,71]. The coexistence of promoter mutations in *TERT* and *BRAF* in PTC is associated with worse clinical outcomes and an increased risk of distant metastasis and poorer survival, indicating a synergistic effect of the two mutations [37,66]. Furthermore, regardless of concomitant gene alterations, *TERT* promoter mutation is predominant among older patients and in larger tumors, as well as being strongly associated with extrathyroidal invasion, angiogenesis, tumor recurrence, and mortality [70,72]. Within a bidirectional pathway, TERT upregulates Wnt/β-catenin pathway, whose impairment is related to many cancer and non-cancer diseases [73] and induces nuclear factor kappa-light-chain-enhancer of activated B cells (NF-κB)-dependent gene expression, such as interleukin (IL)-6, IL-8, and tumor necrosis factor-alpha (TNF-α)—namely, cytokines playing a crucial role in inflammation and cancer progression [72,74,75] (Table 2).

#### 2.1.7. *TP53*

The tumor suppressor gene *TP53*, which controls different cellular processes, including cell cycle progression, DNA repair, apoptosis, and cellular senescence, is the most frequently mutated gene detected in cancers [34,76,77]. In particular, *TP53* mutations cause loss of p53 function and play a key role in tumor progression [34]. These mutations represent a hallmark of ATC, with a mean prevalence of about 60% (but with frequencies up to 78%) and are also relatively common in OCA and PDTC, while they are uncommon in other histological subtypes [37,78,79]. Importantly, while in ATC *TP53* mutations are frequently associated with *TERT* promoter mutations, they are mutually exclusive in non-anaplastic TC, including distant metastatic PTC [79,80]. *TP53* alterations are tightly associated with aggressive features of TC, but unlike *TERT* promoter mutation, their impact on survival is not independent of tumor histology [81,82] (Table 2).

**Table 2:** General features and frequencies of major gene alterations in thyroid cancer.

Gene	Original Function	Alterations	Frequency in TC	References
*RAS*	Encoding GTP-binding proteins within the MAPK/ERK and PI3K-AKT signaling pathways	Point mutations generate three variants: *NRAS* (the most frequent), *HRAS*, and *KRAS.*	68% in FTC 20–40% in ATC 10–30% in PTC	[35,37,48]
*BRAF*	Encoding a serine-threonine kinase of the *RAF* family, which is activated in the MPAK/ERK pathway	Point mutations: *BRAF V600E* are the most common.	Up to 70% in PTC 20–45% in ATC	[35,37]
*RET/PTC*	Transmembrane receptor-type tyrosine kinase stimulating both MAPK/ERK and PIP3/AKT pathways	Chromosomal rearrangements: *RET/PTC1* and RET/PTC3 are the most common.	20% in PTC (up to 70–80% in radiation-exposed subjects)	[60,61]
*PAX8/PPARγ*	*PAX8* encoding a thyroid-specific transcription factor and *PPARγ* encoding a member of the steroid/thyroid nuclear receptor family	Chromosomal rearrangements	12–60% in FTC up to 16% in PTC	[35,37,64]
*PTEN*	Negative regulator of PI3K/AKT pathway	Point mutations	4–33% PDTC 11–20% ATC 0–2% PTC 0–14% FTC	[68]
*TERT*	Encoding the telomerase reverse transcriptase of the enzyme telomerase	Point mutations in the gene promoter: C228T and C250T are the most common.	10–15% in PTC 17% in FTC	[37,71]
			22% in OCA	
			37% in PDTC	
			40% in ATC	
*TP53*	Encoding the tumor suppressor protein p53	Point mutations are generally located in the exons 5–8.	60–78% in ATC 7–12% in OCA 10% in PDTC	[35,78,79]

Abbreviations: ATC: anaplastic thyroid carcinoma; FTC: follicular thyroid carcinoma; MAPK/ERK: mitogen-activated protein kinase/extracellular signal-regulated kinase; OCA: oncocytic carcinoma of the thyroid; PDTC: poorly differentiated thyroid carcinoma; PI3K-AKT: phosphatidylinositol-3 kinase/protein kinase B; PTC: papillary thyroid carcinoma; TC: thyroid cancer.

## 3. Oxidative Stress: The Role of Oxidases in the Thyroid

Oxidative stress is a phenomenon characterized by an imbalance in the production of free radicals (e.g., superoxide radicals, hydroxyl radicals, singlet oxygen, and peroxynitrite, nitrosoperoxycarbonate) and reactive metabolites (e.g., H_2_O_2_), which produces detrimental effects on biological systems [83,84,85]. ROS and reactive nitrogen species, originated from exogenous (toxic metals, chemical solvents, radiation certain medications, smoking, alcohol, and food) and endogenous sources (mitochondrial electron transport chain—ETC and enzymatic reactions involved in immune cell activation, arachidonic acid metabolism, and phagocytosis) are oxidating agents that, when in excess, prevent a fine control of their concentration from antioxidant systems, including nonenzymatic (reduced glutathione—GSH, ascorbate) and enzymatic molecules (catalase—CAT, superoxide dismutase—SOD, glutathione peroxidase—GPX), thus leading to oxidative modifications of lipids, proteins, and DNA and genotoxic responses, up to cell death [16,85,86,87]. If the mitochondrial ETC is the primary endogenous source of ROS, NOX enzymes, multicomponent complexes located in the plasma membrane, are also able to transfer electrons across the plasma membrane to molecular oxygen, generating ROS [86,88]. NOX family comprises seven isoforms: NOX2 (first discovered in phagocytic cells such as monocytes, macrophages, neutrophils, and eosinophils during the study of the respiratory burst), NOX1, NOX3-5, and dual oxidases DUOX1 and DUOX2, which, although ubiquitously expressed, present tissue-specific distribution patterns and expression levels [86,88]. In particular, NOX enzymes exert fundamental actions in numerous processes, including metabolism, cellular signaling, host defense, transcription, and translational regulation [88]. Indeed, while low or moderate levels of cellular ROS are crucial for maintaining biological functions, oxidative stress has been related to a variety of diseases, including diabetes, cardiovascular disease, neurodegenerative disorders, and cancer [83,84,89].

The thyroid gland, responsible for the synthesis and secretion of TH, namely, triiodothyronine and thyroxine, is particularly exposed to oxidative stress during the process of hormonogenesis [17,90]. In fact, the iodine, once entered into the thyroid through NIS, an integral protein of the basolateral membrane of thyrocytes, which mediates active transport of two Na^+^ for each I^−^ into thyroid follicular cells and then in the follicular lumen, is oxidized and subsequently incorporated into the tyrosine residues of Tg by TPO, a transmembrane protein detectable on the apical site of thyrocytes and a key component in TH biosynthesis, which uses H_2_O_2_ as the final electron acceptor [90,91,92]. After the iodination of tyrosyl residues on Tg to form either monoiodotyrosine (MIT) or diiodotyrosine (DIT) moieties, TPO and H_2_O_2_ catalyze the final reaction, which consists of coupling neighboring DIT and MIT residues to generate the active hormones [90].

DUOX1 and DUOX2, the major H_2_O_2_-generating systems, are subject to different activation by intracellular signaling cascades: while DUOX1 is activated by the protein kinase A, DUOX2 is regulated by the protein kinase C within the phospholipase C pathway [91]. Although dual oxidases are also expressed in other tissues, such as the salivary and rectal gland, gastrointestinal tract, respiratory epithelia, and breast tissue, under physiological conditions, they are both expressed exclusively in the thyroid gland, with the expression of DUOX2 five times as high as that of DUOX1 [93,94]. DUOX1/2 activation requires association with DUOX maturation factors, namely, DUOXA1 and DUOXA2, capable of giving rise to heterodimers with DUOX1 and DUOX2, respectively [93]. Indeed, DUOX2 has been established as the isoform to sustain TH production, while the role of DUOX1 has yet to be fully elucidated although it may compensate for the absence of DUOX2 activity [95]. Interestingly, there is a negative correlation between TPO and DUOXs; thus, increased activity of DUOXs determines inhibition of TPO activity, leading to the oxidative damage of the enzyme due to the increased production of H_2_O_2_, which, less consumed by TPO, causes oxidative stress in the thyroid [94]. Furthermore, DUOX2-TPO interaction at the plasma membrane can prevent the diffusion of H_2_O_2_ on the apical surface of thyrocytes, while iodine inhibits DUOX activity at high H_2_O_2_ concentrations [96].

NOX4, the most abundant NOX isoform, is bound to plasma and intracellular membranes through the subunit p22^phox^, which enhances its ROS-generating activity [96]. Originally identified in the kidney, NOX4 is expressed in all tissues although NOX4-mediated-ROS effects depend on the cell type, while the physiological role of NOX4 in the thyroid is currently unknown [16]. In addition to the plasma membrane, NOX4 is found in different intracellular compartments, such as the endoplasmic reticulum, mitochondria, or the nucleus [93,94]. A regulatory protein, termed polymerase δ-interacting protein 2 and highly expressed in thyrocytes, appears to recruit NOX4 to the nucleus where it can exert effects on gene expression and modulatory actions related to DNA damage signaling and DNA replication and repair [93,94,97]. Unlike DUOX, NOX4 exhibits a unique pattern of ROS production, constitutively generating both H_2_O_2_ and superoxide anion, albeit the latter to a small extent (10% of the total), and its activity is only modulated at the transcriptional level by TSH [93,94,97].

### Oxidative Stress in Thyroid Cancer

As discussed in the previous section, the thyroid is physiologically exposed to high levels of ROS, and increasing evidence supports a relevant role of oxidative stress in all three stages of TC, i.e., initiation, promotion, and progression [17,95]. Of note, the thyroid has an elevated spontaneous mutation rate, up to eight times as high as the liver, as also indicated by the higher levels of 8-oxoguanine (oxoG), a marker of DNA oxidation [95]. In fact, patients with different types of TC (for a total of 82 subjects) had higher serum levels of oxidative stress markers, i.e., total oxidant status and oxidative stress index, than healthy individuals; conversely, serum total antioxidant status was significantly lower [98]. Consistently, serum and red blood cell ROS levels and serum malondialdehyde (MDA), a by-product of lipid peroxidation, were significantly higher in patients with PTC (n = 10) compared with the control group [99]. In addition, SOD and GPX activities were significantly reduced in subjects with diagnosis of FTC (n = 6) and in both PTC and FTC groups, respectively, compared with controls [99]. A previous study also documented that, in PTC tissues (n = 9), the levels of MDA were significantly higher than in multinodular goiters (n = 32) and normal adjacent tissues, whereas SOD activity and concentration of selenium (Se, an essential element for TH metabolism and characterized by antioxidant properties [100,101]) were significantly lower than in nodule tissues, suggesting, along a reduced antioxidant defense mechanism, more pronounced lipid peroxidation in cancerous tissue [102]. A recent study, including individuals with coexisting benign thyroid lesions in one thyroid lobe (BTG) and PTC lesion in the other, showed that PTC lesions had significantly higher levels of ROS and different patterns of expression in genes involved in the antioxidant status balance [103]. In particular, compared with BTG lesions, malignant lesions showed increased expression of GPX1, which encodes the most abundant GPX isoform that catalyzes the cleavage of H_2_O_2_ to H_2_O in the presence of GSH; SOD2, encoding the enzyme responsible for the conversion of superoxide anion to H_2_O_2_ and located in mitochondria; and 8-oxoguanine DNA glycosylase (OGG1), encoding a DNA repair enzyme responsible for the removal of oxoG [103,104]. In contrast, PTC lesions had significantly lower expression of CAT, which encodes for the enzyme that breaks down two H_2_O_2_ molecules into two molecules of H_2_O and one molecule of oxygen [105], and peroxiredoxin 1 (PRDX1) (belonging to a family of cysteine-dependent peroxidase enzymes regulating cellular peroxide and peroxynitrite levels, [106]) than BTG lesions [103]. Overall, these findings confirm the redox imbalance as a hallmark in TC, which is characterized by increased mitochondrial levels of superoxide anion, as evidenced by higher SOD2 gene expression and the inability of thyrocytes to clear peroxides leaked from the mitochondria (reduced expression levels of PRDX1) [103]. At the same time, the decrease in CAT expression prevents GPX1 from effectively removing excess ROS in thyrocytes [103]. Furthermore, oxidative DNA damage caused by the hydroxyl radicals, which are produced during the Fenton reaction between H_2_O_2_ and transition metal ions while playing a crucial role in the initiation of thyroid tumorigenesis and PTC progression, also leads to increased OGG1 expression [103]. Another study investigating the levels of ROS in 50 malignant and benign thyroid lesions and 41 normal tissues confirmed that both H_2_O_2_ generation and GPX activity were higher in tumors than in normal tissues, with GPX activity inversely related to the level of oxidative stress [17]. Moreover, PTCs harboring any mutation manifested significantly higher production of ROS than non-mutated PTCs, and H_2_O_2_ production was significantly and positively correlated with tumor stage and American Thyroid Association (ATA) risk [17]. Recently, Sekhar et al. [107] have demonstrated that GPX4, a crucial regulator of ferroptosis, a nonapoptotic form of cell death in cancer cells [108], is overexpressed in TC, and its inhibition is correlated with activation of ferroptosis, subsequent worse overall survival, and increased DNA damage.

Regarding DUOX1/2, their influence on carcinogenesis is controversial, being epigenetically silenced in lung and hepatocellular carcinoma [109,110], while DUOX2 is expressed at high levels in colon, prostate, and breast cancers [111]. Ameziane-El-Hassani et al. [112] showed that human thyroid cells, exposed to ionizing radiation, upregulated both DUOX1 and its maturation factor DUOXA1 several days following irradiation, in a process probably mediated by IL-13. The same authors also reported that radio-induced thyroid tumors during childhood were characterized by significantly increased levels of DUOX1 gene expression compared with normal thyroid tissues, while in sporadic thyroid tumors, the increase in DUOX1 level was borderline significant, identifying DUOX1 as a major source of radio-induced oxidative stress and, as such, capable of promoting genomic instability and inducing TC [91,112]. Conversely, no differences were observed in H_2_O_2_ levels in sporadic PTC, regardless of previous radiation exposure [113].

In contrast to the limited information on its physiological role in the thyroid, NOX4 has been extensively studied in thyroid carcinogenesis [93]. More than 10 years ago, Weyemi et al. [97] found increased expression of NOX4 and its partner p22^phox^ in PTC, suggesting that this continuously producing H_2_O_2_ system might be involved in the signaling mechanism in TC cells. Muzza and colleagues observed that NOX4 protein expression was higher in all neoplastic lesions than in normal thyroid tissues [17]. Furthermore, the level of NOX expression appears to be inversely related to thyroid differentiation, thus the higher mutation burden reported in ATC and PDTC might result from DNA damage promoted by increased ROS generation induced by NOX4 [19,93]. A comparative analysis of NOX4 at the protein level in 134 thyroid tissues (48 TC tissues, 46 normal adjacent tissues, and 40 nonmalignant thyroid tissues) supported the previous findings, showing a higher NOX level in TC tissues compared with normal tissues [114]. In addition, there was a strong positive relationship between the *BRAF V600E* mutation and NOX4 expression; however, regardless of the mutational status, NOX4 was associated with aggressive features in PTC, probably due to the nuclear and perinuclear localization of this NADPH oxidase, which might promote genomic instability associated with thyrocyte transformation even in nonmalignant diseases [114].

NOX4 can be transcriptionally upregulated by *BRAF V600E*-mutated thyroid cells via the TGF-β/ Small mother against decapentaplegic homolog 3 (Smad3) signaling pathway [19]. Transforming growth factor-beta (TGF-β) is overexpressed in human cancers, including TC, able to modulate various processes, such as cell proliferation, differentiation, apoptosis, and migration and acting both as tumor suppressor in the premalignancy phase and as an invasion and metastasis promoter in the advanced stage of cancer [19,115]. Smad3, which activates or represses target gene transcription in the TGF-β pathway and has a higher expression in thyroid tissue than in most other tissues, is involved in the induction of apoptosis, metastasis, and tumor progression [19,116]. TGF-β is a potent repressor of the expression of pivotal specific-thyroid genes, including NIS. Azouzi et al. [19] demonstrated that BRAF V600E-induced repression of NIS via TGF-β signaling involves NOX-4-dependent ROS generation. Alternatively, physical interaction between PAX8, the master transcriptional factor controlling *NIS* transcription, and Smad3 inhibits PAX8 activity, leading to NIS downregulation [117]. Furthermore, BRAF V600E can silence NIS expression both through hypermethylation of the gene promoter region, as confirmed by upregulation of DNA methyltransferase 1 in *BRAF V600E* PTC thyrocytes compared with surrounding normal thyroid follicular cells, and by inducing histone deacetylation in critical regulatory regions of the NIS promoter [118,119]. Given its crucial role in the treatment of DTCs by administering radioactive iodine (RAI) into tumor cells after initial surgery, NIS suppression results in RAI-refractory TC [116,120] (see Section 4.1 for more details).

NOX4 is also involved in the metabolic adaptation of PTC cells [121]. Hypoxia is a major feature of tumors, leading to the formation of new vessels capable of ensuring an adequate supply of oxygen and nutrients necessary for their rapid growth [122]. *NOX4* is a target of the hypoxia-inducible transcription factor 1 alpha (HIF-1α), the most abundant member of a family of master regulators of hypoxia responses in physiological and pathological conditions [123]. Therefore, overexpression of HIF-1α increases NOX4 expression; on the other hand, NOX4-derived ROS are required for the stabilization of HIF-1α and HIF-2α [122,123,124]. The hypoxic microenvironment of malignant tumors induces a metabolic shift, known as the Warburg effect, from oxidative phosphorylation to glycolysis, which represents a key component in maintaining cell proliferation in cancer [93,122]. In the thyroid, low-oxygen conditions are sustained by NOX4-mediated mitochondrial ROS generation, which stabilizes HIF-1α, which, in turn, can upregulate glycolytic enzymes and promote lactate production [122]. Conversely, under conditions of p22^phox^ deletion, PTC cell proliferation and HIF-1α were significantly decreased, and mitochondrial ROS levels and glycolytic flux were both reduced [122].

Of note, serine-threonine adenosine monophosphate-activated protein kinase (AMPK) is another relevant component related to the hypoxic environment of tumor cells, which, acting as an energy sensor, phosphorylates specific enzymes to ATP production and preserves ATP reserves under energy-starved conditions [121,125]. In particular, increased activation of AMPK in PTC cells promotes an antitumor response with inhibition of cell proliferation and invasion, thus inducing cell death [126,127]. Furthermore, AMPK is activated by mitochondrial ROS, and this activation is, in turn, responsible for the inhibition of ROS production in the mitochondria via the peroxisome proliferator-activated receptor-gamma coactivator (PGC)-1α-dependent antioxidant response, PGC-1α being a transcriptional factor that plays a pivotal role in mitochondrial lifecycle and functioning [128,129]. Although the relationship between AMPK and TC remains to be clarified, studies have demonstrated the ability of AMPK to suppress NOX4 expression and the consequent generation of ROS [130,131].

**Figure 1:**
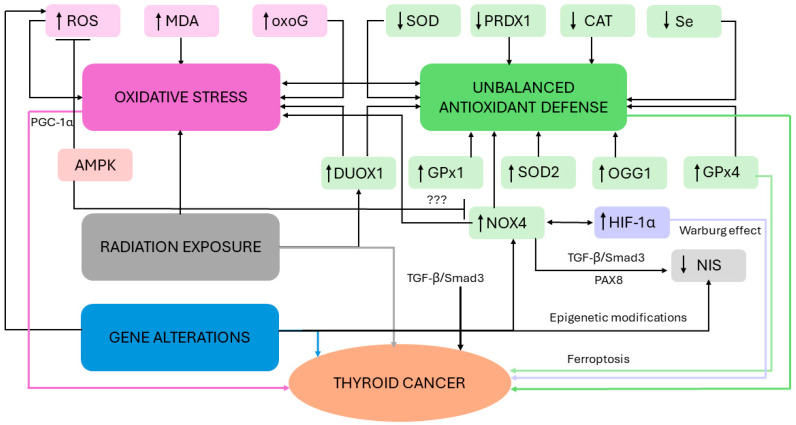
Schematic representation of the mutual relationships and risk factors of the components involved in the generation of oxidative stress and those belonging to the antioxidant defense systems and overall related to the development of thyroid cancer (see text for details). The direction of arrows indicates the increase or decrease in the depicted markers. Abbreviations: AMPK: AMP activated protein kinase; CAT: catalase; DUOX1: isoform belonging to NOX family; GPX: glutathione peroxidase; HIF-1α: hypoxia-inducible transcription factor 1 alpha; MDA: malondialdehyde; NIS: sodium/iodide symporter; NOX: nicotinamide adenine dinucleotide phosphate oxidase; OGG1: 8-oxoguanine DNA glycosylase; oxoG: 8-oxoguanine; PRDX1: peroxiredoxins 1; ROS: reactive oxygen species; Se: selenium; SOD: superoxide dismutase; TGF-β: transforming growth factor-beta.

Overall, oxidative stress is a relevant risk factor for thyroid tumorigenesis and involves a complex network of interconnected multifunctional components that, when associated with TC-harboring mutations, may lead to more severe tumor presentation and progression and poorer prognosis. At the same time, TC shows an unbalanced antioxidant system, which aggravates the disease course, suggesting the need to further investigate the potential targets already identified and to search for others in order to reduce ROS production and ameliorate clinical outcomes in TC (Figure 1).

## 4. Management of Thyroid Cancer

The treatment of TC has seen remarkable advances over the past two decades due to both the improvements in diagnostics and the discovery of genetic markers related to diverse clinical features of TC, including disease recurrence and persistence, which have paved the way to specific indications for surgery and targeted therapies [36,132].

### 4.1. Differentiated Thyroid Cancers

Following the diagnosis of TC by fine-needle aspiration biopsy according to the latest ATA guidelines, the primary treatment of differentiated forms of TC (representing the majority of TC cases) is surgery, with differences based on size and disease burden of tumors [2,30,132]. Indeed, total or near thyroidectomy is recommended in case of TC > 4 cm, extrathyroidal extension or metastasis to lymph nodes or distant sites [30]. On the other hand, for papillary thyroid microcarcinomas (defined as tumors 1 cm or smaller) without evidence of metastasis or local invasion, active surveillance management may represent an alternative to immediate surgery, which should consist of a thyroid lobectomy (unilateral procedure) [30]. For intermediate-sized tumors, the initial surgical procedure can be bilateral or unilateral in the absence of invasive features although total thyroidectomy may be preferable to allow for RAI therapy [30]. Furthermore, preoperative detection of mutations, in addition to providing an indication for total thyroidectomy, may also entail lymphadenectomy in the cervical region [35,133]. In particular, *BRAF V600E*, known as a valuable predictive factor for aggressiveness and worse prognosis in PTC, is significantly associated with cervical lymph node metastasis [134,135].

After total thyroidectomy, RAI therapy, whose principle is based on the ability of NIS expressed in DTC cells to trap radioactive iodine, should be administered according to the initial prognostic indicators for TC, i.e., ATA-risk stratification groups, divided into low, intermediate and high risk, related to death and recurrence, and the postoperative results of serum Tg measurements and neck ultrasonography [30,132,136]. Of note, Tg mutation may promote the development of TC by suppressing the production of TH, making it a valuable marker for the identification of malignant thyroid nodules [137]. Therefore, since Tg is produced by both benign thyroid follicular cells and DTC cells, although with some limitations due to the measurement methods, serum Tg concentration after thyroidectomy is predictive of cancer recurrence and metastases [137,138].

**Figure 2:**
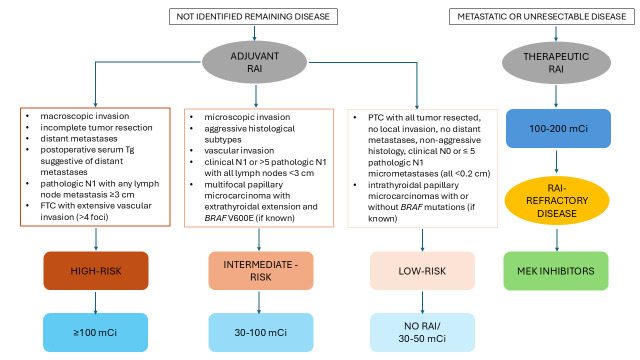
Recommended treatment for differentiated thyroid cancer following thyroidectomy. Risk stratification groups predictive for disease recurrence are based on 2015 American Thyroid Association recommendations [30]. The estimated risk of recurrence has been classified as high (>20%), intermediate (6–20%), and low (≤5%). For the high- and intermediate-risk categories, at least one of the listed characteristics must be present. For the low-risk category, all the listed characteristics must be present. Modified from Filetti et al. [140]. Abbreviations: FTC: follicular thyroid cancer; mCi: millicurie; MEK: mitogen-activated protein kinase/extracellular signal-regulated kinase inhibitors; N0: no evidence of locoregional lymph node metastasis according to the 8th edition of tumor-node-metastasis classification elaborated by the American Joint Committee on Cancer [146]; N1: regional lymph node metastasis according to the 8th edition of tumor-node-metastasis classification elaborated by the American Joint Committee on Cancer [146]; PTC: papillary thyroid cancer; RAI: radioactive iodine; Tg: thyroglobulin.

In a joint proposal between ATA, the European Thyroid Association, the European Association of Nuclear Medicine and Molecular Imaging, RAI should be used with the aims of (i) eliminating residual benign thyroid tissue (remnant ablation) and facilitating follow-ups, improving serum Tg levels and the quality of further imaging studies and/or effectiveness of therapies; (ii) irradiating suspected but unidentified remaining disease (adjuvant treatment) and improving disease-specific and disease-free survival; (iii) destroying known residual or recurrent disease (treatment of known disease), reducing recurrence and increasing progression-free and overall survival [30,136,139] (Figure 2). While low activities (30–50 millicurie—mCi) are usually sufficient for remnant ablation, high activities (≥100 mCi) are required for RAI treatments [139]. In particular, based on 2015 ATA guidelines, low-risk patients—subjects without extrathyroidal invasion, distant metastasis or subtype aggressiveness—are not recommended to undergo RAI ablation although it can be safely applied with 30–50 mCi [30,140] (Figure 2). Nonetheless, the decision for or against the postoperative use of ^131^I in low-risk patients should be made based on individual factors, including the presence of comorbidities, tumor features, and the risk of adverse events [136,141]. On the other hand, for high- and intermediate-risk of recurrence groups, RAI treatment should be administered at high activities (≥100 mCi and 30–100 mCi, respectively [140]) and personalized on an individual basis [136,141] (Figure 2). Notably, to optimize therapy, ^131^I administration should be initiated after TSH stimulation (cut-off > 30 mU/L), by withdrawing levothyroxine for 3–4 weeks, which, however, induces hypothyroidism, or by administering exogenous recombinant TSH [136].

Although RAI therapy has been shown to successfully improve DTC survival after surgery by up to 90%, ionizing radiation may also lead to increased ROS production and consequent DNA damage, as demonstrated by the stable presence of chromosome aberrations within 1 and 3 months after administration of low doses of ^131^I compared with baseline [133,142]. Lipid peroxidation, with significantly higher serum MDA levels, can also be detected in DTC patients undergoing RAI therapy, albeit only after a few days after therapy but no longer after one year [143].

In recent years, one of the most important challenges has been to develop medications to restore RAI uptake in RAI-refractory patients [16,132]. It has been estimated that up to approximately 65% of patients with metastatic or unresectable locoregional disease lose the ability to uptake ^131^I, resulting in RAI resistance [16,144]. MAPK/ERK kinase (MEK) inhibitors (lenvatinib, sorafenib) may represent a valuable therapy for clinically progressive or symptomatic RAI-refractory metastatic DTC [132,144]. Indeed, as discussed in the previous sections, MAPK and PI3K/AKT pathways are the two major activated carcinogenic pathways in DTC and mutations in oncogenes such as *BRAF*, *RAS*, *RET*, activate the MAP kinase signaling pathway, thereby reducing NIS expression. Therefore, inhibition of these pathways may restore iodide metabolism-related gene expression and, consequently, iodine uptake capacity, and enhance sensitivity to ^131^I therapy [132,145] (Figure 2).

### 4.2. Anaplastic Thyroid Cancer

Although it represents less than 2% of all TC cases worldwide, ATC is one of the most aggressive and rapidly growing malignancies, characterized by a poor prognosis with a median survival rate of 6 months and 20% after 12 months from diagnosis. [147,148]. ATC preferentially affects elderly subjects (age > 60 years) and, in percentages ranging from 8% to 80% of cases, may derive from preexisting DTC, suggesting that dedifferentiation processes can be involved in the generation of ATC [147,149]. All cases of ATC are diagnosed at stage IV, with only 10% of patients presenting ATC confined to the thyroid [147]. In patients with the local and locoregionally confined disease (IVA/IVB ATC), overall accounting for around 50% of ATC cases, a total or near-total thyroidectomy and central node dissection surgery should be followed by external beam radiotherapy (EBRT) since ATC does not respond to RAI therapy, with or without concomitant chemotherapy drugs, including anthracyclines (doxorubicin), platinum analogs (cisplatin or carboplatin), and taxanes (paclitaxel or docetaxel), to potentiate the effects of radiation [144,147,150]. The multimodal strategy, applied with the aim of reducing the growth rate of the neck mass and associated with an improvement in overall survival, substantially worsens the quality of life (QoL); therefore, it should be considered within a multidisciplinary discussion to ensure real benefits for selected patients [144,148]. For most patients with stage IVC and many with stage IVB, who survive months or weeks, priority should be symptom control [144,150]. Chemotherapy may be given to patients with unresectable or advanced disease; however, this treatment is highly toxic, presents low response rates, and patients may develop chemoresistance [144,148,150]. In subjects with tumors harboring *BRAF V600E*, targeted therapies may be appropriate—in particular, a combination therapy with the *BRAF* inhibitor dabrafenib and the MEK inhibitor trametinib, which has good tolerability and appears to improve long-term survival [148,151,152]. Activation of PI3K/AKT/mTOR, with mTOR being a protein kinase that controls cellular homeostasis through regulation of proliferation, migration, metabolism, autophagy, and survival processes, occurs in approximately 30–35% of patients with ATC [132,153]. Nevertheless, the efficacy of everolimus, a PI3K/AKT/mTOR inhibitor, has been disappointing in the treatment of ATC [154,155]. Conversely, the use of immunotherapeutic agents may represent a revolutionary choice in the field of anticancer therapies for their action in fighting the tumor by re-establishing the immune system [148]. ATC cells often upregulate programmed death-ligand 1 (PD-L1), which, in addition to cell membrane, is localized in the extracellular space or nucleus where it promotes immune evasion by binding to its receptor PD-1 expressed by T cells, B cells, dendritic cells, and monocytes, and tumorigenesis [132,156,157]. The administration of immune checkpoint inhibitors such as spartalizumab, pembrolizumab, atezolizumab, and anti-PD-1 antibody, alone or in combination with agents targeting specific mutations (e.g., lenvatinib, cobimetinib, and vemurafenib), have yielded promising results, with improved survival and even complete and long-term remission in ATC patients [158,159,160].

## 5. Vitamin C: General Features

Vitamin C, a water-soluble molecule also known as ascorbic acid or ascorbate, cannot be produced endogenously in humans due to inactivating mutations in the gene encoding L-gulonolactone oxidase, an enzyme involved in the final phase of vitamin C biosynthesis process; therefore, the uptake of this micronutrient occurs only through the diet, with fruits and vegetables and their juices as major contributors, or through supplementation [20,21,23,161]. With an absorption rate of 80% at the gastrointestinal level, vitamin C is transported as ascorbate in the plasma, which is considered the primary indicator of body stores, and then distributed to all tissues although the highest levels are detectable in the pituitary glands, adrenal glands, eye lens, and brain [161]. Of note, ascorbate tends to accumulate in circulating cells, i.e., neutrophils, lymphocytes, monocytes, and platelets, with a concentration related to its intake that parallels the relationship between plasma ascorbate concentration and vitamin C intake in adults [161].

The function of vitamin C in biological systems depends on its ability to change the redox state; thus, ascorbic acid, the reduced form, can lose one electron, generating the ascorbyl radical, while the loss of the second electron gives rise to dehydroascorbic acid (DHA), which represents only 1% of plasma vitamin C and can be recycled to ascorbic acid by thioredoxin reductase, dehydroascorbate oxidoreductase, and 3-α-hydroxysteroid dehydrogenase or undergo catabolism in the liver and kidney and be mainly excreted in the urine [20,162,163]. Sodium-ascorbate co-transporters SVCT1 and SVCT2 are responsible for the intestinal absorption and renal reabsorption and cellular accumulation of ascorbic acid, respectively, while DHA transport can be mediated by some isoforms of glucose transporters (GLUT1, GLUT2, GLUT3, GLUT8, and GLUT10) [163].

The biochemical property of donating electrons makes ascorbic acid a potent antioxidant at micromolar concentrations, capable of scavenging ROS and protecting biomolecules from oxidative damage [20,23,162]. On the other hand, vitamin C can also exert pro-oxidant effects in the presence of transition metal such as iron within the Fenton reaction though which ascorbic acid contributes to ROS generation by donating an electron to ferric ion (Fe^3+^) to regenerate ferrous iron (Fe^2+^), which, in the cytosol or in the mitochondria, exists as labile pools and reacts with H_2_O_2_, leading to the production of the hydroxyl radical [23]. Ascorbic acid participates as a cofactor in eight enzymatic reactions, namely, those related to collagen synthesis, carnitine production, the functioning of the central nervous system (through participation in the synthesis of neurotransmitters norepinephrine and epinephrine), and synthesis of nitric oxide [164,165].

**Figure 3:**
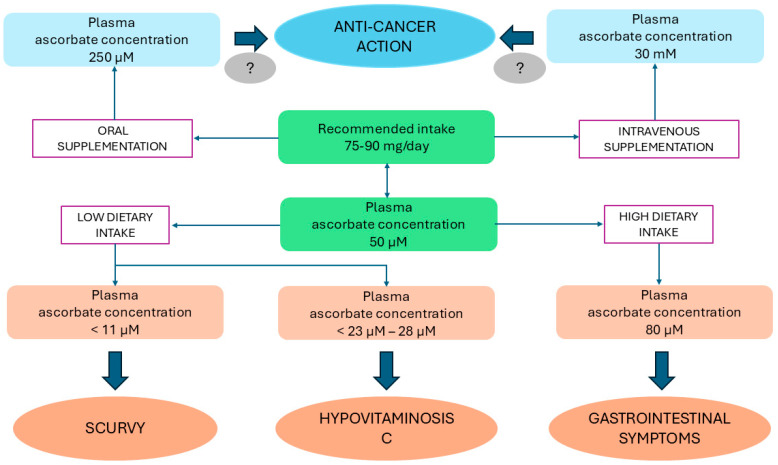
Relationships between dietary intake and supplementation, plasma concentration, and effects of ascorbate in humans.

The current recommended daily dietary allowance of vitamin C by the Institute of Medicine is 90 mg/day for adult men and 75 mg/day for adult women in order to maintain a near-maximal neutrophil concentration with minimal urinary excretion of ascorbate [164], The European Food Safety Authority (EFSA) Panel on Dietetic Products, Nutrition and Allergies established an average requirement of 90 mg/day for men and 80 mg/day for women, based on the amount of vitamin C lost and an adequate plasma ascorbate concentration of 50 µmol/L under fasting conditions [161]. A plasma concentration ≤10 μmol/L and a body pool less than 300 mg result in the development of scurvy, characterized by symptoms related to bone and connective tissue disorders, which can be prevented by a vitamin C intake of 10 mg/day [161]. While doses up to 2 g/day are considered safe, excess vitamin C (3–4 g/day, corresponding to a plasma concentration of 80 µmol/L) may cause reversible gastrointestinal symptoms such as diarrhea despite a decrease in the intestinal absorption due to the reduced expression of the sodium ascorbate transporter SVCT1 [21,161,163]. Data from European dietary surveys indicate a mean daily intake of vitamin C between 69–130 mg/day in men and between 68–138 mg/day in women [161,166]. Overall, based on a recent systematic review, in high-income countries, vitamin C deficiency (defined as plasma ascorbate concentration less than 11 μmol/L) may occur in 0.8% to 26% of the adult population, while up to 52% of adults may experience hypovitaminosis C (<23–28 μmol/L) [166] (Figure 3).

### 5.1. Effects of Vitamin C on Cancer

Although initial interest in the possible anticancer effects of ascorbate arose as early as the 1970s, over the last decade, a growing body of research has documented a potential role of vitamin C in counteracting the development and progression of cancer both in vitro and in vivo models [167,168,169,170]. Early phase (Phase I and II) clinical trials have confirmed that, when administered intravenously at high doses, vitamin C acts as a potent anticancer agent, exhibiting both efficacy and safety and even enhancing the benefits and reducing side effects of standard cancer therapies (i.e., chemotherapy) [21]. On the other hand, the results of clinical trials remain somewhat controversial and no significant effects of vitamin C on cancer have been definitively demonstrated so far due to the almost complete lack of Phase III trials [21,170,171,172]. Compared with oral vitamin C, which leads to a maximum plasma concentration of 250 μM due to limited gastrointestinal absorption, intravenous vitamin C can produce a 100-fold increment of circulating ascorbate levels, up to 30 mM [173] (Figure 3). The systematic review by Fritz et al. [173], which included a total of 37 studies (2 randomized controlled trials—RCTs, 15 uncontrolled trials, 6 observational studies, and 14 case reports) using vitamin C doses ranging from 1 g to more than 200 g ascorbic acid per infusion (generally administered 2 to 3 times weekly), suggests that high-dose (≥5 g) intravenous vitamin C, in combination with other therapies, shows safety and efficacy. In particular, data from 1 RCT suggest that vitamin C, added to paclitaxel/carboplatin (chemotherapy combination), prolongs the time to relapse in ovarian cancer and reduces the side effects of chemotherapy, while those provided by Phase I/II trials and observational studies, although inconclusive due to the intrinsic limitations of the study design and the small sample size, are indicative of an improvement in QoL and, probably, survival and a decrease in disease-related symptoms as well as chemotherapy-associated adverse effects in advanced-stage pancreatic cancer [173]. Case reports results indicated the possibility of cancer remission associated with high doses of vitamin C [173]. A subsequent systematic review of 34 studies (5 RCTs, 12 Phase I/II trials, 6 observational studies, and 11 case reports [174] evaluating the effects of oral (range 1 g/day for 4 days—10 g/day for 28 days) or intravenous alone or combined oral and intravenous ascorbate use (1–30 g daily plus 3–100 g per infusion with variable dose intensity), reported no significant improvements in either overall or progression-free survival or QoL in patients with cancer in controlled trials and a possible improvement in QoL or reduction of symptoms related to concomitant chemotherapy in non-randomized studies when ascorbate was administered intravenously. Furthermore, although ascorbate was generally well tolerated, some signs of potential serious toxicity were occasionally reported in case of intravenous administration. As in [173], the studies included were highly heterogeneous in terms of study design, ascorbate delivery methods, concurrent therapies, and patient characteristics and outcomes analyzed, together with a general paucity of well-designed RCTs [174]. Van Gorkom et al. [175] systematically reviewed 19 trials investigating patients of all ages and sex and any cancer type and the effects of vitamin C supplementation, administered intravenously or orally or in a mixed delivery mode, alone or in combination with other standard cancer treatments, on predefined outcomes. Some studies showed that vitamin C appears to exert beneficial effects on overall survival, clinical response, QoL, and performance status although efficacy is greater for vitamin C administered intravenously than for oral vitamin C, suggesting that oral supplementation may not produce a sufficient dose to promote a possible effect [175]. In particular, in elderly patients with acute myeloid leukemia and treated with decitabine, a chemotherapy medication that upregulates ten-eleven-translocation (TET), methylcytosine dioxygenases having a key role in DNA methylation and being less active in these patients, vitamin C potentiates this effect [126]. The findings for other types of cancer are less clear, probably because of the poor quality of evidence from most studies that were not double-blind [175]. A systematic review including 21 studies (5 of them were RCTs), in which vitamin C was administered orally of intravenously in patients with various malignancies, reported no significant effects of oral vitamin C applied alone or in combination with tumor therapy in the treatment of cancer or in the relief of symptoms [176]. Intravenous ascorbate, when applied as a single therapy, had no beneficial effect except for some improvement in QoL although with conflicting results observed in small, low-quality studies, while vitamin C combined with conventional tumor therapy was associated with improvement in survival, disease progression and performance status, reduced tumor sizes, and pain relief in low-evidence research [176]. Within a comprehensive review, Böttger et al. [24] confirmed previous findings, showing that vitamin C used in monotherapy (four studies) is safe and highly tolerated at doses up to 3 g/kg, corresponding to a maximum plasma ascorbate concentration of up to 49 mM, with rare reports of adverse effects, and can alleviate pain and improve QoL in palliative care. In the included clinical studies (n = 12) evaluating the effects of combined vitamin C with other anticancer therapies, vitamin C was generally administered at doses of ≥1 g/kg/ or ≥75 gr infusion at least 3 times per week (high dose corresponding to a plasma ascorbate concentration ≥20 mM) or ≥10 g whole body (medium dose). All studies reported good toxicity profiles and favorable trends in the control of disease and response rates, but without providing significant evidence of ascorbate efficacy except for metastatic stage IV pancreatic cancer treated with gemcitabine (a chemotherapy drug) and erlotinib (a targeted anticancer cancer medication) combined with vitamin C [24].

Importantly, the potential antitumor effects of vitamin C may vary depending on the type of cancer [22]. An umbrella review assessing the level of evidence in the association between vitamin C and cancer outcomes, including 57 meta-analyses, estimated a significantly decreased incidence of bladder, breast, endometrial, esophageal, gastric, pancreatic, and prostate cancers, cervical neoplasms, glioma, lung, renal cell carcinoma, and total cancer related to the highest dietary/unknown source vitamin C intake compared with the lowest [177]. Additionally, for breast cancer, vitamin C was associated with a reduced risk of recurrence and specific- and all-cause mortality [177]. In contrast, nonsignificant associations were measured between vitamin C intake and incidence of colon and colorectal cancers and non-Hodgkin lymphoma [177]. However, in addition to the generally low quality of the studies included, it cannot be ruled out that the observed effects could depend on the entire set of antioxidants present in fruit and vegetables, the main sources of vitamin C [177]. Recently, the first multicenter Phase III RCT exploring the ability of intravenous high-dose vitamin C to enhance the effects of therapy in patients with metastatic colorectal cancer receiving chemotherapy (FOLFOX ± bevacizumab) with respect to the chemotherapy-only group, found that the progression-free survival of the experimental group was not significantly different from that of the control group, and the objective response rate and overall survival in the two groups were comparable [172]. Of interest, the subgroup RAS-mutated participants receiving vitamin C and chemotherapy showed a longer progression-free survival than those treated with chemotherapy alone [172].

Finally, consistent evidence indicates that oncology patients, in addition to having lower mean plasma vitamin C levels, may commonly present with hypovitaminosis C and ascorbate deficiency (reviewed in [178]). Altered vitamin C status in cancer patients can be attributed to changes in metabolism and signaling pathways related to oxidative and inflammatory processes inherent in the disease, as suggested by increased levels of lipid peroxidation products (e.g., MDA) and pro-inflammatory mediators (e.g., TNF-α, TGF-β, NF-κB, IL-10, and cyclooxygenase 2) [178,179]. Furthermore, low plasma status appears to be more prevalent in patients undergoing chemotherapy or immunotherapy that generate systemic oxidative stress, while the end of treatment leads to a recovery in vitamin C content [178,180,181]

In summary, although a large number of preclinical studies have shown encouraging antitumor effects of vitamin C and some indications from Phase I/II clinical trials may suggest the possibility of improved prognosis in patients receiving high-dose intravenous vitamin C supplementation, the overall low quality of studies conducted so far prevent us from establishing the real efficacy of ascorbate on cancer patients, both as monotherapy and in combination with other therapies. Despite the confirmed high tolerability and safety of vitamin C, robust clinical studies represent a mandatory choice to provide decisive insights into the real antitumor effects of this relevant, high-promising therapeutic option.

### 5.2. The Relationship between Vitamin C and Thyroid Cancer: The Epidemiological Evidence

To date, a limited number of studies have evaluated the effects of vitamin C in patients with TC (Table 3). Two of them have explored the association between dietary vitamin C intake and the risk of TC. The first dated study, which included 399 subjects with histologically-confirmed diagnosis of TC and 617 controls (recruited in hospital setting and with acute, neoplastic, nonhormone-related diseases), observed a borderline significant inverse association between the highest quartile of vitamin C intake (≥225 mg/day) compared with the lowest one (<113 mg/day) and the risk of TC (Odds Ratio—OR = 0.72, 95%CI: 0.5–1.1) after adjusting for age, sex, study center, history of benign thyroid disease and total caloric intake [182]. When data were separately analyzed by sex, an inverse relationship was only observed in females (OR = 0.72 comparing the highest quartile vs. the lowest quartile) but not in males (OR = 1.38) [179]. No significant association was found when considering subjects diagnosed with PTC and FTC separately [182]. Notably, when the model was also adjusted for beta-carotene intake, which strongly protects against TC, the small evidence of an inverse association between the highest quartile of vitamin C consumption and TC risk was further reduced [182]. Therefore, if the results suggest that since fruits and vegetables are the main sources of vitamins, beta-carotene or other associated components could be solely responsible for the protective effect; however, the possibility of recall bias and misclassification cannot be excluded since the information on micronutrient consumption was retrieved through a non-validated questionnaire based on a limited number of food items and with questions only on their frequency [182]. A subsequent case-control study, aimed at evaluating the association between fruit and vegetable intake and risk of TC, enrolled 111 women with histologically confirmed TC and 115 women with benign nodules or adenomas and a corresponding number of age-matched controls who were administered a quantitative food frequency questionnaire based on 121 food items [183]. The study reported that both case groups had lower vitamin C consumption than the corresponding control group although the differences were not significant [183]. Unlike [182], almost all cases were interviewed before diagnosis, and therefore, recall bias between cases and controls was likely not substantial [183]. Within a large prospective study of 482,807 participants to the National Institutes of Health—American Association of Retired Persons Diet and Health Study (NIH-AARP), O’Grady et al. [184] estimated risk associations between dietary micronutrient intake and TC. Assessment of intake was based on a dietary questionnaire including questions on the frequency and portions sizes of 100 food items, while subjects with incident TC were identified through linkage of the NIH-AARP cohort with state cancer registries and National Death Index [184]. After the adjustment for potential confounding factors (age, sex, race, education, calories, physical activity, body mass index, smoking status, vitamin E, beta-carotene, and folate), the authors reported a significant association between the highest quintile of vitamin C intake vs. the lowest quintile and increased risk for TC [Hazard Ratio—HR = 1.46, 95%CI: 1.05–2.4, *p* trend < 0.01) [184]. In addition, dietary intake of vitamin C was significantly associated with risk of PTC, while no significant relationships were instead observed by sex, considering only subjects with FTC [184]. The authors attempted to explain these surprising findings by hypothesizing that participants in the highest quintile of vitamin C consumption, characterized by higher education and physical activity and lower caloric intake, had higher healthcare utilization and, consequently, a higher probability of being diagnosed with TC, while controlling for these factors in the analyses may not have included all aspects of a healthy lifestyle [184].

A set of studies have investigated the potential protective role of vitamin C against the side effects of RAI, which, as discussed in Section 3, is currently the gold standard for DTC treatment after surgery. The absorption of ^131^I, even at low activity, into the salivary glands through NIS, promotes salivary gland dysfunction [185]. RAI-induced sialadenitis, characterized by pain and swelling of the salivary gland, is detected, as an acute form, in 2–67% of patients and, based on recent data, frequently occurs unilaterally in both at the level of the parotid and submandibular glands [186]. Chronic sialadenitis may develop after months and years after the first appearance, with an incidence between 11 and 43% and, together with xerostomia, subjective sensation of dry mouth, which also leads to dysphagia, taste loss, difficulties in chewing and speaking and compromises the QoL of patients undergoing RAI therapy [186,187]. On the other hand, stimulation by vitamin C or other acidic substances following RAI treatment may accelerate the elimination of ^131^I from the salivary glands by improving salivary flow [185]. An early prospective, single blind RCT divided 72 consecutive patients recently diagnosed for PTC or FTC and treated with 100 mCi ^131^I after total thyroidectomy into four groups based on the time elapsed between the end of RAI therapy and the start of vitamin C oral intake (100 mg every 4 h, except during nocturnal rest, for 6 consecutive days) [185]. The authors did not document significant differences in salivary absorbed dose as well as cumulative salivary activities during the first 24 h from ^131^I administration among the four groups [185]. This result can be attributed to the ability of acid stimulation to increase the amount of ^131^I (markedly elevated in the blood immediately after RAI administration), by increasing blood flow, which may partially or completely compensate for the washout effect [185]. A recent study retrospectively analyzed 872 postoperative patients with a diagnosis for DTC and divided them into a 2 and 24 h groups based on the time of initiation of oral vitamin C supplementation (100 mg every hour except during nighttime sleep for 5 consecutive days) after receiving RAI for the first time and reported a significantly lower incidence of both acute (4.78% vs. 15.22% of damage, *p* < 0.001) and chronic salivary gland injury (18.26% vs. 26.09% of damage, *p* = 0–043) in the 2 h group compared with the 24 h group [188]. Furthermore, clinical symptoms related to salivary gland damage were significantly lower in the 2 h groups than in the 24 h group (20.0% vs. 37.4%, *p* < 0.001) overall, indicating that, at this time point, the effect of vitamin C on increasing secretion of salivary glands is greater than that on enhancing blood perfusion although it cannot completely prevent salivary gland damage [188]. Of interest, while no differences were observed between the 2 h and 24 h groups in the concentrate and secretion functions in the parotid and submandibular glands following RAI therapy, when comparing the salivary concentration index of the two submandibular glands after treatment with ^131^I, they were significantly lower than those before treatment in both groups, suggesting the limited action of vitamin C on RAI-induced impaired salivary gland function [188]. In a recent RCT, 89 DTC patients who underwent total thyroidectomy and received the first treatment with 100 mCi ^31^I were divided into three groups, one treated with vitamin E, the second with vitamin C (100 mg every 2 h, for a total of 600–800 mg for 3 days) 2 h following RAI therapy, and the latter with combined vitamin C and supragingival scaling, a procedure to remove plaque and treat periodontitis, and the three groups were compared with regard to functional indices of salivary glands assessed one day before and within one month after receiving RAI [189]. Unlike vitamin E, which showed significant radioprotection effects on parotid excretion function, vitamin C alone did not influence salivary gland functional parameters, while the combined use of ascorbate and supragingival scaling was associated with an increase in the uptake index of bilateral parotid glands and excretion rate of the left parotid gland [189]. Furthermore, serum amylase concentration, a valuable marker of salivary gland damage following irradiation (the pancreatic enzyme is almost unchanged after radiotherapy), was significantly reduced in all groups one month after RAI treatment, with the greatest decrease observed in the subjects undergoing supragingival scaling, which, therefore, appears as a promising therapeutic option for protecting salivary glands from effects of RAI [189,190]. Tong et al. [191] evaluated the effects of vitamin C (administered at the same dose reported in [189]), vitamin E and Se, alone or as combined therapy in 69 DTC postoperative patients diagnosed with DTC and randomly divided into three groups after initial treatment with 100 mCi ^131^I [191]. Patients treated with vitamin C and vitamin E had a significantly higher excretion fraction of the left parotid gland compared with that before treatment [191]. The group of subjects receiving Se and vitamin C presented an even better picture than those treated with Se alone, with an improvement in the excretion function of the parotid glands and the uptake and excretion parameters of the submandibular glands when compared with those measured before treatment, suggesting that Se and vitamin C exert a synergistic effect [191].

Two studies have explored the effects of vitamin C supplementation on RAI-induced oxidative stress (see also Section 4). In a prospective clinical study, 40 DTC patients scheduled to undergo 100 mCi ^131^I after total thyroidectomy were divided into two groups, namely, the intervention group treated with oral supplementation of 2000 mg of vitamin C along with vitamin E and Se for 21 days prior to RAI therapy and the control group that received no treatment [192]. Plasma 8-epi-Prostaglandin F2α (8-epi-PGF2α), a product of arachidonic acid peroxidation and used as a quantitative and reliable marker of systemic oxidative stress (it is stable and its levels are not affected by diet) [193], increased significantly after ^131^I treatment in both groups (with a greater percentage among controls); however, the control group had a significantly higher 8-epi-PGF2α concentration than the intervention group before and both at 2 and 7 days after receiving RAI [192]. Of note, 10% of patients receiving supplements reported side effects although it cannot be excluded that they were due to the acute toxicity of RAI treatment [192]. In a subsequent RTC, 58 DTC patients who received 150 mCi ^131^I were divided into a control group and three other groups based on the different start and end times of oral vitamin C administration (500 mg every 8 h for 48–96 h) [194]. While blood MDA concentration was significantly higher among controls than in the intervention groups with a significant increase in all groups 48 h following RAI administration, SOD activity did not significantly change in any group [194]. Differences were observed for GSH concentration, which was significantly higher 48 h following RAI therapy in Group 4 (vitamin C received for two days prior to RAI) than in controls and with significant mean variations between the control group and Group 3 (vitamin C administered over time between 48 h before and 48 h after RAI) and Group 4 [193]. CAT activity significantly increased in all groups 48 h after treatment, with the highest value measured in the control group and the lowest in Group 3 [194]. Therefore, if these findings confirm the increase in oxidative stress following RAI treatment, the significant change in MDA, GSH, and CAT between the control group and Group 4 indicates that vitamin C exerts a radioprotective and scavenger effect when administered before RAI [194]. In contrast, vitamin C supplementation used after RAI had a rather a mitigating effect, as suggested by the smallest increase in MDA and the slightest increase in CAT among the four groups [194].

In summary, some evidence suggests that orally administered vitamin C, especially in association with other supplements (vitamin E, Se), could exert a protective action against salivary glands damage and oxidative stress caused by RAI treatment. However, studies performed so far were generally based on a limited number of patients diagnosed for only DTC and with a short follow-up. Therefore, future RCTs, involving a larger number of participants also undergoing multiple treatments with ^131^I and treated with different doses and durations of vitamin C alone and at different administration times from the start of RAI therapy, would be useful to provide more knowledge on the real effects of vitamin C on salivary glands. On the other hand, increased dietary consumption of vitamin C does not seem to be associated with a reduced risk of TC, but data are scarce and conflicting, and this relevant issue should be investigated in large-scale multicenter studies (Table 3).

**Table 3:** Summary of characteristics of studies investigating the association between vitamin C and its effects on thyroid cancer.

Study Design—Study Period	Country	Population	Overall Effect	Limitations—Pitfalls	Reference
Case-control 1986–1992	Italy	A total of 399 subjects (291 F, 108 M, age 16–72 years) diagnosed with any TC type within 2 years prior to interview. 691 control subjects (427 F, 190 M, age 16–74 years)	Weak evidence of inverse association between increasing quartiles of dietary vitamin C intake and risk for TC	Possibility of recall bias related to food frequencies. Selection bias of controls who may have had different dietary habits.	[182]
Case-control 2008–2010	Republic of Korea	Only women: 111 pairs of malignant TC (90% PTC) and controls (mean age 45.9 and 45.2 years, respectively) and 115 pairs of benign cases and controls (mean age 46.8 and 46.5 years, respectively). Patients were asked to report their food intake over a 12-month period, starting 3 years prior to the time of the interview.	No significant differences in vitamin C intake between patients with and without TC and controls	Small sample size and no possibility to evaluate differences by TC type and risk factors such as radiation exposure.	[183]
Prospective cohort 1995–1996	United States	A total of 482,807 subjects (287,944 M, 194,863 F, age 50–71 years). 592 incident PTC or FTC cases (257 M, 335 F).	Significantly positive association between increasing quintiles of dietary vitamin C intake and risk for TC	Only patients with DTC were included. There was a possibility of residual confounders related to a healthy lifestyle/consciousness.	[184]
Prospective, single -blind RCT	China	A total of 72 patients aged ≥ 18 years were diagnosed with PTC and FTC and treated with 100 mCi for 4–6 weeks after total thyroidectomy. Subjects were divided into 4 groups: 18, 18, 19, and 17 of them started vitamin C administration at 1,5,13, and 25 h after RAI therapy, respectively.	No significant effect of vitamin C administration at any time on salivary absorbed ^131^I of TC patients undergoing RAI.	Only patients with DTC were included. There was a lack of investigation on the salivary function and symptoms after RAI.	[185]
Retrospective clinical 2014–2020	China	A total of 872 patients were diagnosed with DTC treated for the first time with RAI after thyroidectomy. Subjects were divided into 2 groups: 324 (mean age 45.06 years) starting vitamin C administration at 2 h after RAI therapy and 548 (mean age 45.29 years) starting vitamin C administration at 24 h after RAI.	Significantly higher protective effect of vitamin C on the salivary glands at 2 h than at 24 h after receiving ^131^I.	Only patients with DTC were included. Follow-up ended at 6 months. Inability of vitamin C to restore salivary gland function after ^131^I was observed.	[188]
RCT 2019–2021	China	A total of 89 patients (35M, 54F, age 22–68 years) with DTC and tumor-node-metastasis at stages I/II underwent total thyroidectomy. Subjects were divided into 3 groups: Treatment with 0.2 gr daily vitamin E for 5 weeks initiated 1 week before and continued until 4 weeks after RAI therapy (n = 30, mean age 43 years); Treatment with vitamin C (n = 30, mean age, 44.1 years); Treatment with vitamin C and supragingival scaling (n = 29, mean age 42.3 years).	No significant effects of vitamin C alone on salivary gland parameters but only combined with supragingival scaling	Only patients with DTC were included. There was a limited number of subjects for each group. The follow-up period was too short. No measurement of salivary gland amylase was made either in the initial period or in the long term after RAI treatment. Patients with multiple ^131^I treatments were not included in the study.	[189]
Prospective RCT August 2019– November 2021	China	A total of 69 postoperative patients with DTC Were divided into 3 groups: Treatment with 200 mg/day vitamin E from 1 week before to 4 weeks after RAI treatment + 100 mg every 2 h (600–800 mg/day) vitamin C from 2 h before to 3 days after RAI treatment (n = 21); Treatment with 200 µg/day Se 2 h to a month after RAI treatment (n = 23); Treatment with 100 mg every 2 h (600–800 mg/day) vitamin C from 2 h before to 3 days after RAI treatment + 200 µg/day Se 2 h to a month after RAI treatment (n = 25).	Significant improvement in salivary gland functions following treatment with combined therapy of vitamin C and Se.	Only patients with DTC were included. There was a limited number of subjects for each group. The follow-up period was too short.	[191]
Prospective clinical May 2013–March 2014	Brazil	A total of 40 patients with DTC undergoing thyroidectomy (33 F, 7 M, age 18–60 years) were divided into 2 groups: the first 20 subjects were without supplements; the following 20 subjects were treated with 2000 mg vitamin C, 1000 mg vitamin E, and 400 µg Se for 21 days before RAI treatment.	Attenuation of RAI-induced oxidative stress by supplementation of vitamin C and other antioxidants.	No conclusions were made on the real effect of vitamin C. There were possible side effects of antioxidant supplementation.	[192]
RCT March–July 2017	Iran	A total of 45 patients (age 23–78 years) were diagnosed with DTC and underwent 150 mCi RAI. Subjects were divided into 4 groups: RAI + no vitamin C administration (n = 16); RAI + vitamin C immediately for 48 h from immediately after RAI (n = 14); RAI + vitamin C from 48 h before RAI to 48 h after RAI (n = 14); RAI + vitamin C only for 48 h before RAI.	Antioxidant effect of vitamin C against serum oxidative stress induced by RAI. Radioprotective action of vitamin C to be preferred to its mitigating effects.	Only patients with DTC were included. Small sample size.	[194]

Abbreviations: DTC: differentiated thyroid cancer; FTC: follicular thyroid cancer; mCi: milliCurie; PTC: papillary thyroid cancer; RAI: radioactive iodine; RCT: randomized controlled study; Se: selenium; TC: thyroid cancer.

#### The Mechanism Underlying the Association between Vitamin C and Thyroid Cancer

In the last decades, various biological processes have been proposed to explain the antitumor effects of ascorbate in vitro and partly in vivo although the sensitivity of cancer cells may vary depending on the type of cancer and the tumor dependence on specific pathways (see [23] for more details). In addition, some experimental studies have specifically investigated the potential mechanisms underlying the action of vitamin C in TC [162,195,196,197]. Overall, these biological mechanisms can be summarized as follows:

1. As reported above in the text, ascorbate promotes pro-oxidants effects in the presence of H_2_O_2_ and labile iron, which is primarily located in the cytosol in small pools bound to low-affinity ligands, generating ROS through the Fenton reaction [168,170]. Increased levels of ROS are a hallmark of cancer, stimulating cell proliferation and affecting genetic stability; however, excessive amounts of ROS may lead to cell death. Intravenous ascorbate can kill tumor cells even in the absence of Fe^2+^ by inducing the generation of extracellular H_2_O_2_ via spontaneous autoxidation. On the other hand, the tumor microenvironment is enriched in labile Fe^3+^, which can promote the oxidation of ascorbate resulting in the production of DHA, Fe^2+^, and superoxide anion, the latter converted to H_2_O_2_ by SOD. Furthermore, extracellular H_2_O_2_, reacting with extracellular or intracellular Fe^2+^, generates hydroxyl radicals, responsible for selective toxicity to cancer cells [23]. Indeed, tumor cells show greater sensitivity to ascorbate than normal cells due to increased levels of labile iron pools resulting from impaired oxidative metabolism in mitochondria, characterized by increased H_2_O_2_ and superoxide anion [168,198]. In addition, H_2_O_2_ generated from ascorbate may disrupt intracellular Fe–S bonds, thus leading to a further increase in labile iron pools in cancer cells [198]. However, it is unlikely that the Fenton reaction, demonstrated in vitro, occurs in vivo at physiological concentrations of Fe^2+^ and Fe^3+^, which are also normally chelated by metal-binding proteins [170,199,200]. Moreover, the hypoxic tumor microenvironment may not be favorable for the generation of H_2_O_2_, which is strictly dependent on the oxygen level [169].
2. As discussed in Section “Oxidative Stress in Thyroid Cancer”, tumor cells exhibit an increased rate of glycolysis, which allows for enhanced cell survival and proliferation. Upregulation of the glucose transporter GLUT1 by *KRAS* or *BRAF* mutations may further contribute to the glycolytic phenotype. Due to its structural similarity to glucose, DHA is transported into cells mainly via GLUT1 and GLUT3, and this may explain the selective toxicity of high-dose vitamin C observed in cultured colorectal cells harboring *KRAS* or *BRAF* mutations [169,201]. Indeed, the reduction of DHA to ascorbate inside cells results in the consumption of GSH and the production of ROS, which, in turn, leads to the inactivation of glyceraldehyde 3-phosphate dehydrogenase (GADPH), with consequent inhibition of glycolysis and ATP production, up to cell death [23,200]. Alternatively, GADPH activity can be suppressed through activation of poly(ADP-ribose) polymerase, which plays a crucial role in DNA repair and preservation of genome integrity, thereby depleting cellular stores of GADPH cofactor NAD^+^ [168,201,202]. It should be noted, however, that decreased glycolysis-related markers in *KRAS* and *BRAF* cells can be equally induced by H_2_O_2_-mediated toxicity [169]. Additionally, the half-life of DHA at physiological pH is approximately 1.5 h; therefore, its concentration in vivo may not be sufficient to compete with glucose for transport via GLUT1 [169].
3. Hypermethylation of DNA and histones are recognized as hallmarks of cancer that prevent tumor cells from responding to the signals and effects of chemotherapy [203]. Ascorbate may act as an epigenetic modulator by enhancing the reaction of TET proteins (TET1, TET2, TET3), DNA hydroxylases responsible for DNA demethylation through the conversion of 5-methylcytosine (5 mC) to 5-hydroxymethylcytosine, promoting antitumor effects (see Section 5.1) [170]. *TET2* frequently exhibits loss-of-function mutations in hematologic malignancies resulting in overall DNA hypermethylation [23,203]. Ascorbate administration, which induces TET activity by recycling Fe^3+^ to Fe^2+^, restores TET2 phenotypes and DNA demethylation, enhances chemosensitivity, and drives an increased expression of tumor suppressor genes and genes critical for cell differentiation [23,204,205]. Patients with acute myeloid leukemia have genomes with mutual exclusivity in mutations in *TET* and in genes encoding isocitrate dehydrogenase 1 and 2 (*IDH1/2*), the latter promoting aberrant DNA methylation through increased production of 2-hydroxyglutarate that inhibits the hydroxylation of 5 mC and induces disease [206]. Vitamin C treatment, in addition to TET activation, reduces proliferation induced by neomorphic mutations in *IDH* and promotes differentiation of myeloid progenitor cells [24,205]. Vitamin C is also required for the normal function of Jumonji C domain-containing histone demethylases, which catalyze histone demethylation by producing highly reactive oxoferryl species that, through substrate hydroxylation, induce loss of the methyl group [206].
4. Vitamin C is also a cofactor of collagen prolyl-4-hydroxylases (P4Hs), which belong to a superfamily of iron and 2-oxoglutarate-dependent hydroxylases and catalyze the post-translational hydroxylation of peptidyl–proline residues to 4-hydroxyproline in the presence of oxygen [169,207]. In particular, HIF-P4Hs, enzymes located in the cytoplasm and nucleus, together with asparagine hydroxylase (also known as factor-inhibiting HIF—FIH) are responsible for the regulation of HIF1, a key transcription factor expressed in various types of solid cancers where it modulates both angiogenesis and glycolytic system (see Section “Oxidative Stress in Thyroid Cancer”) [170]. HIF1 is a heterodimer composed of two subunits, the oxygen-regulated HIF-1α in the cytoplasm and the constitutively expressed HIF-1β in the nucleus. Thus, while under normal oxygen conditions, HIF-1α activity is downregulated by HIF-P4H and FIH, and under conditions of hypoxia and oxidative stress or ascorbate deficiency, typical of tumors, HIF-P4H and FIH are inhibited and HIF-1α induces gene transcription, neoangiogenesis, tumor growth, and progression, as well as lack of responsiveness to RAI and chemotherapy [23,115,208]. Interestingly, HIF activation, a phenomenon occurring during the process of carcinogenesis, enhances the cytotoxicity of vitamin C on a variety of cancer cell lines by promoting the uptake of DHA (whose production from ascorbate is increased in tumors) via GLUT1, which is also a transcriptional target of HIF [209].
5. Contrary to the above findings, pharmaceutical doses of vitamin C inhibit cell proliferation and induce apoptosis in TC cells regardless of *BRAF* mutation status at physiological glucose levels, and this effect is mediated by increased cellular ROS levels [195]. The same authors also observed a reduction in TC volume and weight in xenograft and transgenic mice after intraperitoneal injection of vitamin C [195]. In combination with vemurafenib (PLX4032), a selective oral inhibitor of BRAF V600E associated with improved overall survival of patients with metastatic melanoma, vitamin C synergistically suppresses the proliferation and induces cell apoptosis and cycle arrest of *BRAF*-mutated TC cells, potentiating the effects of chemotherapy drug used as monotherapy [196,210]. Furthermore, a combination therapy of vitamin C and PLX403 enhances tumor growth reduction in vivo compared with either agent alone [196]. In contrast to metastatic melanoma, PLX4032 appears to have mild efficacy in colorectal cancer and TC due to transient inhibition of MAPK signaling, which ultimately leads to drug resistance [211]. Consistently, treatment with PLX403 monotherapy results in increased ERK and AKT phosphorylation in *BRAF*-mutant TC cells, while the combination of vitamin C and PLX403 inhibits the ROS-dependent feedback activation of MAPK/ERK pathways and AKT increase (see next point) [196].
6. As reported in Section 2.1, MAPK/ERK and PI3K/AKT represent the two major pathways implicated in thyroid tumorigenesis and TC progression; therefore, they are ideally the most suitable therapeutic target in TC. Regardless of *BRAF* mutation status, vitamin C treatment can substantially inhibit the activity of both pathways in a dose-dependent manner through a ROS-dependent decrease in total AKT levels, as well as ERK and AKT phosphorylation [195]. Two mechanisms have been hypothesized to explain the inhibition of ERK phosphorylation: (a) in *BRAF* mutated cells, vitamin C blocks the production of ATP (see point 1 in this subsection), which frequently donates the phosphate group to protein kinases; (b) in *BRAF* wild-type TC cells, vitamin C-induced ROS generation results in reduced release of epidermal growth factor (EGF), which, binding to the EGF receptor (EGFR), leads to ERK phosphorylation. Therefore, suppression of EGF release and phosphorylation inhibits MAPK/ERK signaling and ERK phosphorylation [195]. EGFR is a tyrosine kinase receptor whose mutations and overexpression can promote a vast number of pro-oncogenic biological processes, such as cell proliferation and motility, adhesion, angiogenesis, inhibition of apoptosis, and metastasis [212]. Furthermore, EGFR overexpression in TC cells seems to be responsible for the progression toward a dedifferentiated phenotype presenting with poorly differentiated and anaplastic areas [213]. The action of vitamin C on AKT instability is instead mediated by the upregulation of mitochondrial E3 ubiquitin protein ligase 1 (MUL1), which promotes ubiquitination of AKT via a ROS-dependent pathway [195].
7. Ferroptosis, an iron-dependent type of cell death highly related to ROS and with lipid peroxidation as its hallmark, has distinctive morphological and biochemical features compared with other regulated forms of cell death (apoptosis, autophagy, cuproptosis, necroptosis, and pyroptosis) [214,215,216] (see also Section “Oxidative Stress in Thyroid Cancer”) Vitamin C can significantly block the growth of ATC cells by activating ferroptosis through a dramatic increase in ROS-induced MDA levels and downregulation of GPX4, in dose- and time-dependent manners [197,215]. GPX4, considered a key inhibitor of phospholipid peroxidation, catalyzes the conversion of lipid peroxides into the corresponding alcohols, also contributing to the maintenance of the integrity of the cell membrane and regulates the metabolism of iron by preventing it from participating in the Fenton reaction, which is the crucial step for ferroptosis [217]. The effects of vitamin C observed in ATC cells are probably mediated by ferritinophagy, a novel autophagy process associated with ferroptosis, which plays a crucial role in a variety of physiological processes such as cell differentiation and erythropoiesis and whose impairment is linked to several diseases, including cancer and hemochromatosis, the latter due to iron overload [218]. Ferritinophagy involves ferritin degradation in a process promoted by the nuclear receptor coactivator 4, thereby resulting in the release of iron into the cytoplasm, which, when in excess, can generate ROS and cause cell death [216,218]. Thus, ferritinophagy promoted by vitamin C leads to the release of iron, which, as reported in the previous sections, may give rise to lipid peroxidation via the production of ROS in the Fenton reaction [197].
8. Vitamin C administration to selected PTC-derived cells, all carrying *TERT* promoter mutations and other specific mutations/rearrangements, causes a significant increase in cell death in all cell lines [25]. In contrast, a slight increase in apoptosis only occurs in cells with *BRAF V600E* and *TP53* mutations, which also show significantly higher production of ROS and decreased GSH/oxidized glutathione ratio after vitamin C treatment compared with corresponding untreated cells [25]. In cells harboring *BRAF V600E* (with concomitant or not *TP53* mutations), exposure to high-dose vitamin C leads to a reduced cysteine/cystine ratio, with cysteine being the major component of GSH and participating in numerous redox reactions [25,219]. The described redox imbalance, triggered by an increase in vitamin C-induced ROS production, is likely responsible for a metabolic deterioration characterized by reduced glucose uptake and glycolysis, depletion of nicotinamide adenine dinucleotide (NAD^+^), a key molecule regulating energy metabolism, resulting in impaired tricarboxylic acid cycle (TCA), and increased levels of upstream metabolites in glycolysis and TCA [25,220,221]. Alternatively, high vitamin C concentration may inhibit the activity of chrome-b5-oxidoreductase-3 (Cyb5R3), which, under normal conditions, catalyzes the conversion of ascorbyl free radical to ascorbate using NADH, thus contributing to maintaining the NAD^+^/NADH ratio in cells [25,218]. Conversely, the downregulation of Cyb5R3 results in a decline of NAD^+^/NADH ratio, compromising mitochondrial respiration and, consequently, ATP production [222].

**Figure 4:**
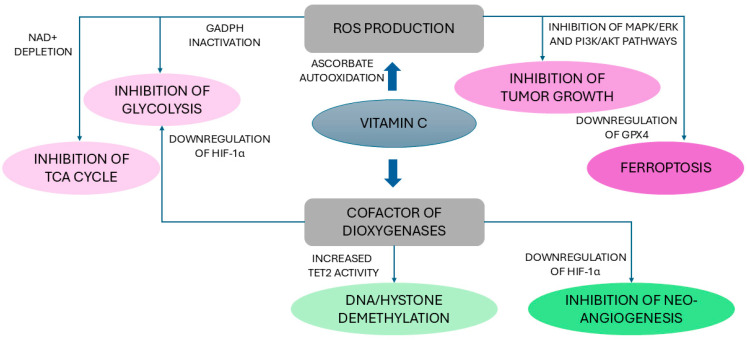
The proposed mechanisms for anticancer effects of vitamin C (see text for more details). Abbreviations: GADPH: glyceraldehyde 3-phosphate dehydrogenase; GPX4: glutathione peroxidase 4; HIF-1α: hypoxia-inducible transcription factor 1 alpha; the MAPK/ERK: mitogen-activated protein kinase/extracellular signal-regulated kinase; NAD^+^: oxidized nicotinamide adenine dinucleotide; TCA: tricarboxylic acid; PI3K/AKT: phosphatidylinositol-3 kinase/protein kinase B. ROS: reactive oxygen species.

Overall, a large amount of data is suggestive of anticancer effects of vitamin C in vitro models and xenograft animals, and the mechanisms proposed can be traced back to two different types, namely, high dose-ascorbate redox mechanisms and as a cofactor of 2-oxoglutarate–dependent dioxygenases (Figure 4). The hypotheses formulated need to be confirmed in vivo to design appropriate clinical studies and identify the most suitable dose of ascorbate to be administered in combination with conventional therapies and, therefore, potentially improve the care and QoL of patients with TC and, in general, with cancer.

## 6. New Strategies for Vitamin C Intake through Food

The potentially positive effects of diet on the thyroid are mainly associated with the consumption of certain types of foods, including fruit, which holds interesting bioactive compounds, such as vitamin C, beta carotene, flavonoids, limonoids, folic acid, and dietary fibers, all of which exhibiting important antioxidant characteristics that could also protect against TC [223,224,225]. Indeed, the Mediterranean diet, characterized by a large consumption of fruit, vegetables, legumes, fish, complex carbohydrates, and extra virgin olive oil, which are rich in various oligo-elements and vitamins, has an overall beneficial effect on preventing thyroid diseases, including TC [226]. Conversely, the use of organophosphate pesticides, widely applied in agriculture, has been associated with a significantly increased risk of TC [227].

As discussed in Section 5, vitamin C consumption could decrease the likelihood of developing TC, however, with a supposedly increased efficacy when the source of this component comes directly from food intake, probably due to the synergistic effects brought by the presence of several other antioxidants [182,183,223,224,225].

This is particularly true when food items are cultivated in a natural fashion, without making use of pesticides, heavy metals, and other compounds, which have potentially harmful effects on human health.

### Beneficial Edible Compounds Production and Sensory Characteristics

As stated, the first essential characteristic edible compounds must have in order to deliver all the potential benefits that are normally contained in them is represented by being rid of specific contaminants that could negatively affect sensory features, as well as their beneficial properties for the consumer. In this regard, several initiatives have been started at various levels, for example, to foster the so-called circular economy, like the ones under the European Green Deal umbrella, known as the New Circular Economy Action Plan (CEAP) [228], and the Farm to Fork strategy [229]. Such initiatives aim to reduce the use of artificial fertilizers and the loss of food and nutrients and scale up the amount of organic farming and water reuse; however, this new policy has also put new challenges when it comes to the identification and management of pollutants, yet not fully considered until being present in food research, including personal care products, heavy metals, plant protection products, and per- and polyfluoroalkyl substances, like those being present in poorly treated sewage sludges [230]. Other typical cases of such new threats include the elevated dioxin level due to the recycled mineral oil used in the production of pig feed, retrieved in pig meat in Ireland as of 2008 [231], or the dramatically high concentrations of polybrominated diphenyl ethers, coming from recycled fish meals, found in British fish feed as of 2013 [232]. Therefore, such threats must be taken into account alongside the traditional highways for contamination, which are still actual, especially considering the framework of less wealthy countries where the regulations are not particularly strict and the safety checks are often scattered [233,234].

**Figure 5:**
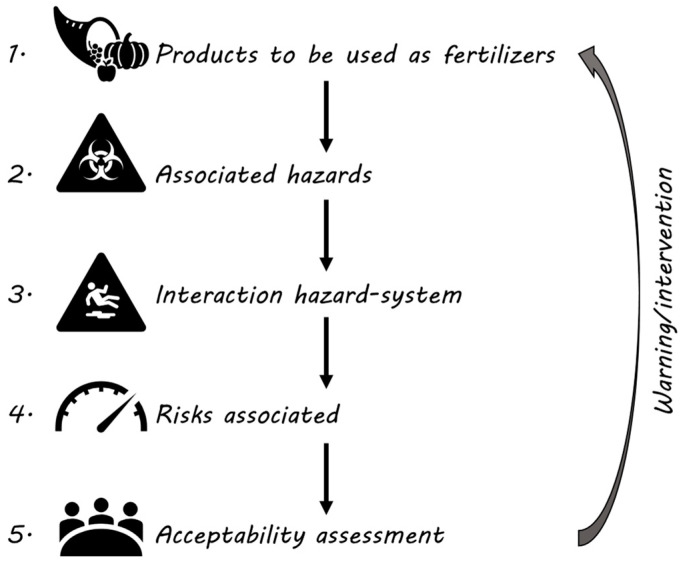
Risks related to food production in the age of circular economy.

With such premises, the production of agricultural compounds to be conveyed to the consumers’ table includes the assessment of some main principles to be taken into account carefully, as displayed in Figure 5.

Aside from such evaluation, sensory analysis should be conducted in order to ensure another pivotal characteristic for a given edible compound, which is represented by its acceptability by the consumers, the real end users of the product.

Sensory analysis is normally conducted to determine the main sensory characteristics of a given compound as perceived by the consumer, represented by the panelist in that case. More specifically, the sensory analysis is performed using a group of experienced tasters, who are properly trained to provide semi-quantitative results in terms of the organoleptic features they perceive within the food compounds, through structured questionnaires they are asked to fill. Within this scenario, a pivotal role is performed by the panel leader [235], who must be experienced enough to get the panel rid, as much as possible, of biases and outliers of judgment. However, this traditional approach presents two main criticisms. At first, panelists, although experienced and well-grounded in the activities related to sensory analysis, do not represent the average consumer, in terms of their approach to food and their abilities when it comes to subtle chemosensory differences between compounds (see, for example, [236]). Second, relying on a set of questionnaires a volunteer is asked to provide explicit answers to, such methodologies carry on significant judgment biases due to difficulties of successful administration [237]; therefore, the integration of traditional analysis methods with a new perspective is key to the success of the sensory analysis applied to food overall [238]. To the best of our knowledge, the sensory analysis applied to products that are rich in vitamin C, including citrus, was performed using only traditional approaches, either instrumental or human-based [239,240,241,242,243]; however, the inclusion of implicit methods would overtake current limitations of this approach, further enriching this important analysis, ultimately leading to enhanced acceptance by the end users and maximizing the likelihood of vitamin C supplementation through foods for those who are in need of that and refuse to eventually undertake specific edible compounds due to personal preferences.

## 7. Conclusions

While accumulating evidence indicates that vitamin C, if applied intravenously and in high doses, may have beneficial effects in oncology patients who often have low or even deficient plasma ascorbate levels, while reducing the toxicity associated with standard antitumor therapy and with good tolerability for patients, as for TC, a limited number of studies, both experimental and clinical, have been conducted so far. Although with some inconsistencies, there are promising indications that vitamin C oral supplementation may protect against adverse effects (sialadenitis, oxidative stress) of RAI therapy, which, however, needs to be confirmed in further RCTs with a larger sample size and not only limited to patients with differentiated forms of TC. At the same time, while several hypotheses regarding the mechanisms of action of ascorbate in cancer treatment have been recently proposed, new in vivo models are warranted to verify these effects in conditions mimicking the human organism. Furthermore, the cytotoxic effects of vitamin C observed in TC cells, together with the possible mechanisms underlying these actions, shed light on the potential use of this nutrient as a treatment choice for TC, alone or in combination with conventional therapies, as it would appear to enhance their effects. Therefore, the current evidence paves the way for investigations aimed at providing further insights into this relevant issue at both experimental and clinical levels and at identifying the most suitable dose of vitamin C in cancer treatment in general, and specifically in TC for which it could serve as an adjuvant therapy to improve the QoL of patients and the efficacy of traditional therapies. This new information could also form the basis for evaluating the correlations between ascorbate treatment and selected biomarkers to be analyzed with omics assays in large-scale clinical studies and open new frontiers for TC care, which is still the most frequent endocrine neoplasia nowadays. Finally, the acceptability of edible compounds, which are rich in vitamin C, is key to the success of the intake of such components, and in this regard, a multimodal, multidisciplinary analysis, involving sensory panels and consumers, is essential to the good outcome of this action.

## Figures and Tables

**Table 1:** Main features of malignant thyroid carcinomas based on 2022 WHO classification.

Type	Genetic Alterations	Subtypes	Invasion	Prognosis
FTC	Mostly *RAS* mutations	Minimally invasive Encapsulated angioinvasive Widely invasive	Invasion of the tumor capsule or the blood vessels	Generally excellent but depending on the extent of the invasion
IEFV-PTC	*RAS* mutations	Minimally invasive Encapsulated angioinvasive Widely invasive	Invasion of the tumor capsule (or adjacent tissue) or the blood vessels	Generally excellent but depending on the extent of the invasion
PTC	Mostly *BRAF* mutations *TERT* promoter mutations *RET/PTC* rearrangements	Infiltrative follicular Tall cell Columnar cell Hobnail Diffuse sclerosing Solid/trabecular Oncocytic Warthin-like	Perineural and lymphatic invasion Lymphatic and vascular invasion Diffuse lymphatic infiltration Vascular invasion Lymphatic infiltration Lymphoplasmacytic invasion	Generally excellent in the absence of vascular invasion Possibility of aggressive clinical course in diffuse sclerosing and solid subtypes
OCA	Mitochondrial DNA mutations in ETC Complex I subunit *RAS* mutations *TERT* promoter mutations	Minimally invasive Encapsulated angioinvasive Widely invasive	Capsular, focal, or extensive vascular invasion	Depending on distant metastasis at diagnosis
DHGTC	Mostly *BRAF* mutations *TERT* promoter mutations *TP53* mutations	-	Vascular, lymphatic, perineural, and extrathyroidal invasion	Intermediate
PDTC	Mostly *RAS* mutations *TERT* promoter mutations *TP53* mutations	-	Vascular, lymphatic, perineural, and extrathyroidal invasion	Intermediate
ATC	*BRAF* mutations *RAS* mutations *TERT* promoter mutations *TP53* mutations	Squamous cell carcinoma	Local and extrathyroidal invasion	Poor

Abbreviations: ATC: anaplastic thyroid carcinoma; DHGTC: differentiated high-grade thyroid carcinoma; ETC; electron transport chain; FTC: follicular thyroid carcinoma; IEFV-PTC: invasive encapsulated follicular variant papillary thyroid carcinoma; OCA: oncocytic carcinoma of the thyroid; PDTC: poorly differentiated thyroid carcinoma; PTC: papillary thyroid carcinoma.

## Data Availability

No new data were created.

## Abbreviations

AMPK	Adenosine monophosphate-activated protein kinase
ATC	Anaplastic thyroid cancer
BRAF	v-raf murine sarcoma viral oncogene homolog B1
CAT	Catalase
DHA	Dehydroascorbic acid
DIT	Diiodotyrosine
DTC	Differentiated thyroid cancer
DUOX	Dual oxidases
EFV-PTC	Encapsulated follicular variant papillary thyroid cancer
EGF	Epidermal growth factor
EGFR	Epidermal growth factor receptor
FIH	Factor-inhibiting hypoxia-inducible transcription factor
FTC	Follicular thyroid cancer
GADPH	Glyceraldehyde 3-phosphate dehydrogenase
GLUT	Glucose transporters
GPX4	Glutathione peroxidase
GSH	Glutathione (reduced form)
H_2_O_2_	Hydrogen peroxide
HIF-1α	Hypoxia-inducible transcription factor 1 alpha
IDH	Isocitrate dehydrogenase
IL	Interleukin
MAPK/ERK	Mitogen-activated protein kinase/extracellular signal-regulated kinase
mCi	Millicurie
MDA	Malondialdehyde
MIT	Monoiodotyrosine
mTOR	Mammalian target of rapamycin
NAD^+^	Nicotinamide adenine dinucleotide (oxidized form)
NAPH	Nicotinamide adenine dinucleotide phosphate (reduced form)
NIFTP	Noninvasive follicular thyroid neoplasm with papillary-like nuclear features
NIS	Sodium/iodide symporter
NOX	Nicotinamide adenine dinucleotide phosphate oxidases
NF-κB	Nuclear factor kappa-light-chain-enhancer of activated B cells
OGG1	8-oxoguanine DNA glycosylase
oxoG	8-oxoguanine
PAX8	Paired box gene 8
PGC-1α	Peroxisome proliferator-activated receptor-gamma coactivator-1 alpha
PPAR-γ	Peroxisome proliferator-activated receptor gamma
PRDX1	Peroxiredoxin 1
P4H	Prolyl-4-hydroxylase
PTC	Papillary thyroid cancer
PTEN	Phosphatase and tensin homolog
QoL	Quality of life
RAS	Rat sarcoma
RCT	Randomized controlled trial
RET	Rearranged during transfection
ROS	Reactive oxygen species
SMAD3	Mothers against decapentaplegic homolog 3
SOD	Superoxide dismutase
SVCT	Sodium-ascorbate co-transporters
TC	Thyroid cancer
TCA	Tricarboxylic acid
TERT	Telomerase reverse transcriptase
TET	Ten-eleven-translocation
TG	Thyroglobulin
TH	Thyroid hormones
TNF-α	Tumor necrosis factor-alpha
TP53	p53 tumor suppressor
TPO	Thyroperoxidase
TGF-β	Transforming growth factor-beta
TSH	Thyroid stimulating hormone
